# The Role of Xanthan Gum and Hydroxypropyl Methylcellulose in Gluten‐Free Bread: A Study of Physical Characteristics, Texture, and Nutrition

**DOI:** 10.1002/fsn3.71292

**Published:** 2026-01-14

**Authors:** Fetriyuna Fetriyuna, Annisa Nur Salma, Putri Widyanti Harlina, Vira Putri Yarlina, Ratna Chrismiari Purwestri

**Affiliations:** ^1^ Department of Food Technology, Faculty of Agro‐Industrial Technology Universitas Padjadjaran Jatinangor Kab. Sumedang Indonesia; ^2^ Study Center for the Sustainable Development of Agroindustry, Faculty of Agro‐Industrial Technology Universitas Padjadjaran Jatinangor Indonesia; ^3^ Faculty of Forestry and Wood Sciences Czech University of Life Sciences, Prague Praha‐Suchdol Czech Republic

**Keywords:** bread, gluten‐free bread, HPMC, hydrocolloid, XG

## Abstract

The development of gluten‐free bread innovations is driven by increased public awareness of healthy living, the rising number of individuals with celiac disease and gluten allergies, and efforts to reduce dependence on wheat imports by utilizing local commodities. Xanthan Gum (XG) and hydroxypropyl methylcellulose (HPMC) are commonly used hydrocolloids in gluten‐free bread because they have been proven effective in improving gluten‐free bread quality and mimicking the function of gluten in the bread matrix. This literature review aims to evaluate the roles of XG and HPMC in gluten‐free bread based on physical characteristics (specific volume, organoleptic properties, and color), texture, and nutrition. This study employs the Textual Narrative Synthesis method and PRISMA (Preferred Reporting Items for Systematic Reviews and Meta‐Analyses) technique, resulting in 41 selected studies after a comprehensive literature assessment. The review findings indicate that the addition of XG and HPMC to gluten‐free bread can enhance specific volume, sensory quality, and texture characteristics. The type of hydrocolloid, flour or starch used, water content, and the addition of various ingredients in the formulation influence the physical, textural, and nutritional properties of gluten‐free bread. The use of these hydrocolloids offers a solution for the food industry in producing high‐quality gluten‐free bread.

## Graphical abstract

This literature review investigates how effectively hydroxypropyl methylcellulose (HPMC) and xanthan gum work to enhance the texture, volume, and sensory characteristics of gluten‐free bread. The review comes to the conclusion that these hydrocolloids greatly improve bread quality, based on an analysis of 41 studies. The type of flour, water content, and formulation components all have an impact on these hydrocolloids' effects.

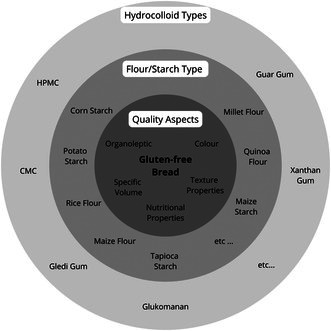

## 1 Introduction

Gluten‐free products are developing along with the increasing number of people with diseases related to gluten, such as wheat allergy, celiac disease, and non‐celiac gluten sensitivity (Brouns et al. 2013; Golley et al. 2014; Reilly 2016). People with celiac disease cannot consume products that contain gluten, as it may cause damage to the intestinal wall and interfere with the absorption of nutrients (Green and Cellier 2007; Harris 2011). Most bakery products are made from wheat flour, which contains gluten protein (Surono et al. 2017). The absence of gluten in bread results in low gluten‐free bread development volume (Muna et al. 2023). Gluten plays a role in protein‐starch interactions that give bread dough certain viscoelastic properties and in the stabilization and retention of gas cells during proofing and baking (Ahlborn et al. 2005). Therefore, it is necessary to use alternative ingredients that can replace the function of gluten in gluten‐free bread products.

Hydrocolloids are the most commonly used ingredient to replace gluten (Mir et al. 2016). Commonly used hydrocolloids in the food industry include agar, glucomannan, xanthan gum (XG), hydroxypropyl methylcellulose (HPMC), and pectin (Achmadi 2022). Their incorporation can modify the viscoelastic and rheological properties of dough, resulting in gluten‐free breads with characteristics that are more similar to those made with wheat flour. For example, certain hydrocolloids improve gas retention, moisture binding, and dough stability, which are crucial for bread structure and texture.

Among these, XG and HPMC are the two most commonly used and recognized hydrocolloids compared to other hydrocolloids (Crockett et al. 2011; Hager and Arendt 2013). This is because XG and HPMC are able to produce gluten‐free bread with superior and preferred characteristics. Their superiority lies in consistently producing bread with higher specific volume, softer crumb structure, and improved sensory acceptance compared to other hydrocolloids (Anton and Artfield 2008; Hager and Arendt 2013; Kang et al. 1997). This advantage has led to XG and HPMC becoming the primary focus of numerous studies, further reinforcing their importance in the food industry.

XG is an anionic polysaccharide that is commercially produced from a fermentation process by the bacterium *Xanthomonas campestris* . Structurally, XG consists of a cellulose‐like backbone of β‐(1 → 4)‐D‐glucose units with trisaccharide side chains containing mannose and glucuronic acid, which contribute to its high molecular weight and anionic character (Sworn 2009). These structural features give XG unique rheological properties, such as high viscosity at low concentrations, pseudoplastic (shear‐thinning) flow behavior, and stability over a wide range of pH and temperatures (Mohammadi et al. 2014). In general, XG produces bakery products with increased moisture and volume, higher crumb strength, improved texture, and moisture retention in dough and gluten‐free bread (Sworn 2009).

HPMC is the most commonly used type of hydrocolloid in the manufacture of gluten‐free bread (Crockett et al. 2011; Hager and Arendt 2013). This is because HPMC can produce bread with better specific volume characteristics, softer bread crumb, and superior sensory attributes (Barcenas and Rosell 2005; Guarda et al. 2004; Rosell et al. 2001). HPMC is a semi‐synthetic derivative of cellulose, obtained by substituting hydroxyl groups of the cellulose chain with methyl and hydroxypropyl groups (Li et al. 2013; Sarkar and Walker 1995). The cellulose backbone is typically sourced from wood pulp or cotton linters, making HPMC a plant‐derived hydrocolloid. Its physicochemical properties include water solubility, thermo‐gelling behavior (forming a gel upon heating and returning to solution upon cooling), and the ability to form stable films (Crockett et al. 2011; Hager and Arendt 2013). These properties are particularly advantageous in gluten‐free breadmaking, as HPMC provides viscoelasticity to dough, mimics the gas‐holding capacity of gluten, and produces loaves with higher specific volume, softer crumb texture, and superior sensory attributes (Barcenas and Rosell 2005; Guarda et al. 2004; Rosell et al. 2001).

Bread specific volume is one of the most important visual characteristics of bread products and is a key parameter observed when evaluating bread quality (Hager and Arendt 2013). Another important aspect of gluten‐free bread characteristics is texture. Consumers desire a soft and pliable texture, which is associated with low hardness values (Hager and Arendt 2013). Color is an important visual characteristic in food and is one of the aspects that influence consumer preferences and purchasing decisions. Calle et al. (2020) stated that the color parameter of bread is the first characteristic observed by consumers, thus determining consumer choice and preference. In terms of nutritional content, available gluten‐free products generally do not contain the necessary nutrients, both in quality and quantity, to maintain a balanced diet (Alencar et al. 2012).

This literature review synthesizes current evidence on the role of XG and HPMC in determining the physical properties, textural attributes, and nutritional quality of gluten‐free bread. Emphasis is placed on XG and HPMC as the most extensively applied hydrocolloids, whose effectiveness in improving the overall quality of gluten‐free formulations has been consistently demonstrated.

## 2 Methodology

This research design uses the literature review method, which is a systematic and explicit review method that involves the identification, evaluation, and synthesis of research findings and ideas generated by researchers (Okoli and Schabram 2011). The analysis in this study was carried out descriptively in the form of textual narrative synthesis (TNS). The use of the textual narrative synthesis method can integrate several studies into more homogeneous subgroups and includes quantitative tabulation of research data, which can provide a more thorough description and explanation (Xiao and Watson 2019).

**FIGURE 1:**
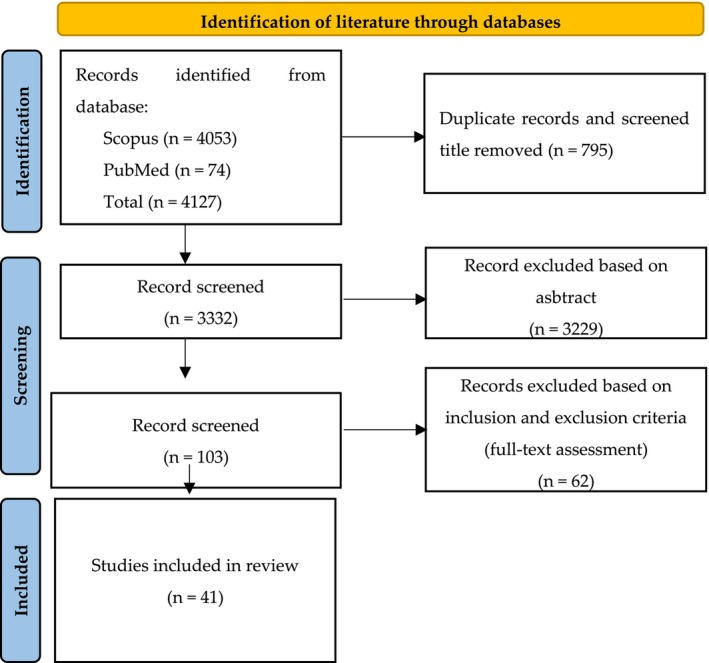
Flowchart example of the systematic review search process based on PRISMA guidelines.

The systematic literature review, utilizing the TNS method, was performed according to PRISMA (Preferred Reporting Items for Systematic Reviews and Meta‐Analyses), which details the process for study selection and exclusion (Figure 1).

The literature sorting process began with a search using keywords on Scopus and PubMed. These searches utilized relevant keywords to identify literature on appropriate topics. Searching the library using the keywords resulted in 4127 libraries, which will then be sorted. Of the 4127 libraries, 795 libraries were eliminated based on the title and the existence of duplicates, so that 3332 libraries remained. Furthermore, 3332 libraries are filtered based on their abstracts. A total of 3229 libraries were excluded, leaving 103 libraries. Then, 103 libraries were screened for full‐text so that the remaining 41 libraries could be included in the discussion of the literature review.

## 3 Results

The development of gluten‐free bread products must consider product characteristics similar to conventional bread made with wheat flour to be accepted by consumers. The quality characteristics that determine consumers' choice of bread products are generally related to their appearance, such as size (volume) of the bread, color (surface color of the bread), shape of the bread, and freshness (related to texture and softness of the crumb) (Dapˇcevi'c‐Hadnađev et al. 2013).

### 3.1 The Role of XG and HPMC in the Physical Characteristics of Gluten‐Free Bread

The specific volume of bread is one of the most important visual characteristics of bread products and is a key parameter observed when evaluating gluten‐free bread quality (Hager and Arendt 2013). McCarroll and de Kock (2017) stated that in gluten‐free bread products, consumers prefer larger bread volumes. The effect of XP and HPMC on the specific volume of gluten‐free bread is presented in Table 1.

**TABLE 1:** Specific volume of gluten‐free bread with addition of XG and HPMC.

Hydrocoloid	Type of flour/starch	Treatment	Specific volume (cm^3^/g)	References
XG	Corn starch (100%)	XG 0.5%	2.24 ± 0.04	Vidaurre‐Ruiz et al. (2019)
XG	Corn starch (80%) + zein (20%)	XG 2%	3.86 ± 0.23	Sadat et al. (2023)
XG	Corn starch 60 g	MF CGM 20 g + XG 5 g	3.25 ± 0.04	Ozturk and Mert (2018)
	Corn starch 70 g	MF CGM 20 g + XG 5 g	3.59 ± 0.07	
	Corn starch 80 g	MF CGM 20 g + XG 5 g	3.01 ± 0.04	
XG	Light buckwheat flour 88.2 g + chia flour 9.8 g	XG 2 g	2.04 ± 0.05	Coronel et al. (2021)
XG	Maize starch (100%)	XG 2%	2.25 ± 0.08	Belorio and Gómez (2020)
XG	Maize starch 100 g + mageu 456 g + sorghum meal fine 169 g + soybean flour 124 g	XG 0.5%	2.86 ± 0.50	McCarroll and de Kock (2017)
XG	Potato starch (100%)	XG 0.5%	2.77 ± 0.05	Vidaurre‐Ruiz et al. (2019)
XG	Proso millet flour (100%)	XG 0.5%	2.25 ± 0.03	Tamilselvan et al. (2022)
XG	Proso millet flour (100%)	XG 1.0%	2.22 ± 0.01	
XG	Proso millet flour (100%)	XG 1.5%	2.16 ± 0.06	
XG	Proso millet flour (100%)	XG 2.0%	2.08 ± 0.05	
XG	Rice flour (100%)	XG 2%	2.60 ± 0.15	Franco et al. (2020)
XG	Rice flour (100%)	XG 1% + CMC 1%	2.43 ± 0.13	
XG	Rice flour (100%)	XG 0.5% + CMC 1.5%	2.48 ± 0.07	
XG	Rice flour (100%)	XG 1.5% + CMC 0.5%	2.64 ± 0.10	
XG	Rice flour (100%)	XG 4%	±1.88	Jang et al. (2018)
XG	Rice flour (100%)	XG 0.25%	±2.5 (× 10^−3^ m^3^/kg)*	Fujii et al. (2023)
XG	Rice flour (100%)	XG 0.50%	< 2.5 (× 10^−3^ m^3^/kg)*	
XG	Rice flour (100%)	XG 0.75%	> 2.25 (× 10^−3^ m^3^/kg)*	
XG	Rice flour (100%)	XG 1.0%	±2.25 (× 10^−3^ m^3^/kg)*	
XG	Rice flour (100%)	XG 2%	1.48 ± 0.03	Belorio and Gómez (2020)
XG	Rice flour (30%) + small broken riceberry flour (70%)	XG 1%	0.47 ± 0.02	Rakkhumkaew et al. (2019)
XG	Rice flour (50%) + maize flour (30%) + quinoa flour (20%)	XG 1.5%	1.78	Encina‐Zelada et al. (2018)
XG	Rice flour (50%) + maize flour (30%) + quinoa flour (20%)	XG 2.5%	1.73	
XG	Rice flour (70%) + buckwheat (30%)	XG 2%	1.9 ± 0.5	Burešová et al. (2016)
XG	Rice flour (95%) + DHF (5%)	XG 0.25%	< 2.5*	Tunç and Kahyaoglu (2016)
XG	Rice flour + corn flour + corn starch (100%)	XG 1%	2.623 ± 0.363	Naji‐Tabasi and Mohebbi (2015)
XG	Rice flour + potato flour + corn starch (100%)	Proportion of XG and G 0.75:0.25	2.7023 ± 0.1436	Sutrisno et al. (2021)
XG	Rice flour 45 g + cassava starch 45 g + soy flour 10 g	Gl 0.25% + XG 0.25%	1.96 ± 0.16	Sciarini et al. (2023)
XG	Rice flour 45 g + cassava starch 45 g + soy flour 10 g	Gl 0.63% + XG 0.63%	1.74 ± 0.09	
XG	Rice flour 45 g + cassava starch 45 g + soy flour 10 g	GG 0.25% + XG 0.25%	2.13 ± 0.18	
XG	Rice flour 45 g + cassava starch 45 g + soy flour 10 g	GG 0.63% + XG 0.63%	1.93 ± 0.16	
XG	Small broken rice flour 46.45 g	XG 1.0 g	±1.20*	Numfon (2017)
XG	Small broken rice flour 46.45 g	XG 1.5 g	> 1.20*	
XG	Small broken rice flour 46.45 g	XG 2.0 g	> 1.10*	
XG	Tapioca starch (60%) + millet flour (40%)	XG 1%	±1.3*	Chakraborty et al. (2020)
XG	Tapioca starch (60%) + millet flour (40%)	XG 1.5%	±1.5*	
XG	Tapioca starch (60%) + millet flour (40%)	XG 2.0%	±1.6*	
XG	Tapioca starch (60%) + millet flour (40%)	XG 2.5%	±1.6*	
HPMC	Colocasia cormles rhizome flour (100%)	HPMC 2%	1.70 ± 0.05	Calle et al. (2020)
HPMC	Corn starch (80%) + zein (20%)	HPMC 2%	6.06 ± 0.42	Sadat et al. (2023)
HPMC	Maize starch (100%)	HPMC 2%	7.58 ± 0.04	Belorio and Gómez (2020)
HPMC	Maize starch (30%) + yellow maize flour (70%)	HPMC 4% + SBF 3%	2.44	Djordjević et al. (2019)
HPMC	Maize starch (30%) + yellow maize flour (70%)	HPMC 4% + AF 3%	3.29	
HPMC	Maize starch (30%) + yellow maize flour (70%)	HPMC 4% + SBF 7%	2.08	
HPMC	Maize starch (30%) + yellow maize flour (70%)	HPMC 4% + AF 7%	3.97	
HPMC	Maize starch 100 g + mageu 456 g + sorghum meal fine 169 g + soybean flour 124 g	HPMC 0.8%	2.89 ± 0.54	McCarroll and de Kock (2017)
HPMC	Proso millet flour (100%)	HPMC 2.0%	2.88 ± 0.07	Tamilselvan et al. (2022)
HPMC	Quinoa flour (27/69%) + grass pea flour (30%) + chestnut flour (42/31%)	HPMC 0.5%	3.11 ± 0.02	Tabrizi et al. (2023)
HPMC	Quinoa flour (27/69%) + grass pea flour (30%) + chestnut flour (42/31%)	HPMC 1.0%	3.40 ± 0.06	
HPMC	Quinoa flour (27/69%) + grass pea flour (30%) + chestnut flour (42/31%)	HPMC 1.5%	3.54 ± 0.09	
HPMC	Rice flour (100%)	HPMC 4% + WPC 4%	1.69 ± 0.04	Srikanlaya et al. (2018)
HPMC	Rice flour (100%)	SFE 0.7% + NE 1.9% + BBG 2.95% + water 100%	3.69	Pérez‐Quirce et al. (2014)
HPMC	Rice flour (100%)	HPMC 2%	3.85 ± 0.39	Zhao et al. (2021)
HPMC	Rice flour (100%)	HPMC 2% + PGA 0.5%	4.26 ± 0.11	
HPMC	Rice flour (100%)	HPMC 2% + PGA 1.0%	4.60 ± 0.22	
HPMC	Rice flour (100%)	HPMC 1% + PGA 0.5%	2.93 ± 0.06	
HPMC	Rice flour (100%)	HPMC 1.5% + PGA 0.5%	4.49 ± 0.03	
HPMC	Rice flour (100%)	HPMC 0.5%	2.45 ± 0.04	Imamura et al. (2021)
HPMC	Rice flour (100%)	HPMC 4%	±4.3	Jang et al. (2018)
HPMC	Rice flour (100%)	HPMC 2%	1.33 ± 0.01	Belorio and Gómez (2020)
HPMC	Rice flour (50%) + millet flour (50%)	HPMC 1% + CRJ 3%	4.49 ± 0.02	Abdollahzadeh et al. (2024)
HPMC	Rice flour (50%) + millet flour (50%)	HPMC 2% + CRJ 3%	3.06 ± 0.00	
HPMC	Rice flour (80%) + soy flour (20%)	HPMC 1.5%	2.61 ± 0.22	Huerta et al. (2016)
HPMC	Rice flour 38 g + corn starch 38 g	WOP 1.5%, + HPMC 1.5%	3.07 ± 0.28	Tufaro et al. (2022)
HPMC	Rice flour 38 g + corn starch 38 g	FOP 1.5% + HPMC 1.5%	3.89 ± 0.32	
HPMC	Rice starch (100%)	WPI 0.37% + HPMC 4.35% + b‐glucan 1%	4.53 ± 0.04	Kittisuban et al. (2014)
HPMC	Unripe banana flour (100%)	Water 52% + UBF‐P 28% + HPMC 6%	4.29 ± 0.16	Hernández‐Aguirre et al. (2019)
XG and HPMC	Colocasia cormles rhizome flour (100%)	HPMC 0.29% + XG 0.21% + GG 0.50%	1.67 ± 0.02	Calle et al. (2020)
XG and HPMC	Rice flour (50%) + maize flour (30%) + quinoa flour (20%)	XG 0.24% + GG 0.60% + HPMC 3.16%	2.57	Encina‐Zelada et al. (2019)
XG and HPMC	Rice flour (52%) + potato starch (36%) + sweet cassava flour (12%)	PDCF 20.00% + HPMC 0.51% + XG 0.51%	1.96 ± 0.02	Ewerling et al. (2020)

*: Data are presented as mean +‐ sd, or +/‐ or < in accordance to results of the cited scientific articles.

Research by Naji‐Tabasi and Mohebbi (2015) showed an increase in the specific volume value of bread made from a combination of corn starch, corn flour, and rice flour with the addition of XG. In line with this, a study conducted by Ozturk and Mert (2018) on gluten‐free bread from corn starch showed that the use of XG increased the specific volume of this bread by 1.03–1.22 times compared to that without the addition of XG.

Meanwhile, in a study conducted by Fujii et al. (2023), the addition of XG caused a decrease in specific volume compared to the control group. This can be caused by the addition of XG, increasing the viscosity of the dough, which prevents the dough from expanding. A decrease in the specific volume of gluten‐free bread with the addition of XG was also obtained from the research of Tamilselvan et al. (2022) and Coronel et al. (2021). This was attributed to the higher dough consistency and resistance that reduced dough development during baking (Numfon 2017).

The effect of the type of hydrocolloid used in gluten‐free bread production largely depends on the physicochemical properties of flour or starch used. This dependence is partly attributed to variations in amylose‐to‐amylopectin ratio and protein content among different starch and flour sources (Horstmann et al. 2016). Belorio and Gómez (2020) compared gluten‐free breads produced from corn starch and rice flour, finding that formulations based on corn starch exhibited a higher specific loaf volume than those based on rice flour when using either XG or HPMC. This difference was attributed to the higher pasting consistency of rice flour relative to corn starch, primarily due to its higher protein content (Martínez and Gómez 2017). According to (Martínez and Gómez 2017), proteins can encapsulate starch granules, thereby modifying their gelatinization kinetics and increasing gelatinization temperature. Furthermore, differences in water‐holding capacity (WHC) contribute to these differences, as starches have lower WHC values than flours (Matia‐Merino et al. 2019).

The molecular interaction between hydrocolloids and starch or flour components is highly specific. Amylopectin exhibits stronger associative interactions with hydrocolloids than amylose, owing to its highly branched molecular configuration. This branching facilitates the formation of a more interconnected three‐dimensional polymer network, thereby enhancing dough viscoelasticity and structural stability (Crockett et al. 2011; Linlaud et al. 2011). XG primarily forms hydrogen bonds with amylopectin molecules, thereby generating a cohesive matrix capable of retaining both water and gas during fermentation and baking. Such interactions are critical for improving crumb texture, gas cell uniformity, and loaf expansion in gluten‐free bread (Barcenas and Rosell 2005).

These findings indicate that the functional performance of hydrocolloids in gluten‐free systems is modulated by the relative proportions of amylose and amylopectin in the base starch or flour. Moreover, granule morphology, surface roughness, and particle size distribution also influence hydrocolloid–starch interactions, ultimately affecting dough rheology and structural development during baking (Bárcenas and Rosell 2006).

Numfon (2017) reported that the incorporation of 1.0–1.5 g of xanthan gum (XG) into gluten‐free bread prepared from small broken rice flour increased the specific loaf volume. However, when the XG content was increased to 2.0 g, a reduction in specific volume was observed. A similar trend was noted by Chakraborty et al. (2020). Studies optimizing hydrocolloid dosage consistently demonstrate that exceeding the optimal concentration leads to reduced loaf volume. This behavior suggests that dough rheology in gluten‐free systems is formulation‐dependent, and that hydrocolloid concentration must be carefully optimized to maintain sufficient viscosity for gas cell stabilization without inhibiting expansion (Nishita et al. 1976).

At elevated levels of XG addition, the reduction in specific volume arises from excessive dough rigidity, which restricts expansion during baking (Numfon 2017). This effect results from extensive hydrogen bonding between the carboxyl groups in XG and hydroxyl groups of water and starch, producing a rigid gel matrix (Chakraborty et al. 2020). When hydrocolloid concentrations surpass the optimal threshold, gas cell coalescence and expansion are hindered because the network becomes excessively rigid and less deformable (McCarroll and de Kock 2017).

Besides XG, HPMC has also been applied in gluten‐free bread formulations. McCarroll and de Kock (2017) reported that HPMC treatment yielded a higher average specific loaf volume compared to XG and guar gum. This finding is corroborated by Zhao et al. (2021), who demonstrated that HPMC increased the specific volume of gluten‐free bread by up to 350%, whereas other hydrocolloids (XG, CMC, PGA, and konjac gum) produced volumes comparable to the control samples.

Sadat et al. (2023) reported that the incorporation of 2% HPMC into gluten‐free bread formulated with 80% corn starch and 20% zein yielded a specific volume of 6.06 mL/g. At the same level of HPMC addition, a markedly lower specific volume (1.70 mL/g) was obtained when using Colocasia cormless rhizome flour as the sole flour source (Calle et al. 2020). These results indicate that the type of flour employed substantially influences the specific volume of gluten‐free bread.

The effect of hydrocolloid addition on the specific volume of gluten‐free bread is closely linked to the hydration level incorporated into the formulation. Belorio and Gómez (2020) reported that, at 100% water addition, gluten‐free bread containing HPMC exhibited more than twice the specific volume of gluten‐free bread containing XG, whereas, at 70% water addition, both formulations achieved similar specific volumes. This difference is attributable to the high water‐binding capacity of XG, which yields a highly viscous dough. In contrast, HPMC‐based doughs maintain a more balanced viscoelastic structure, enabling greater gas retention and expansion. The superior performance of HPMC is linked to its thermo‐reversible gelation behavior during baking, which enhances dough viscosity, strengthens gas cell walls, and minimizes moisture migration, thus improving loaf volume (Crockett et al. 2011).

During baking, the dough undergoes a series of thermo‐mechanical and physicochemical transformations, primarily driven by starch gelatinization and protein denaturation–coagulation. During starch gelatinization, amylose leaching from swollen granules allows interaction with hydrophobic macromolecules, particularly proteins and hydrocolloids (Sciarini et al. 2023).

The inclusion of hydrocolloids enhances dough stability by forming a three‐dimensional viscoelastic matrix that reinforces both the pre‐baked dough structure and the crumb architecture of the final product (Lazaridou et al. 2007). HPMC contains hydroxypropyl and methoxyl functional groups: the former engages in hydrogen bonding with hydroxyl (–OH) groups of starch and water, while the latter exhibits amphiphilic properties, functioning as a surfactant at the starch–gas interface and thereby improving interfacial film strength (Gallagher et al. 2004). During baking, as water evaporates, strong intermolecular interactions occur between HPMC chains, resulting in the formation of a gel that establishes a robust network. This network stabilizes gas cells within the dough, improves gas retention, and ultimately increases loaf volume (Barcenas and Rosell 2005).

### 3.2 The Role of XG and HPMC in the Sensory Characteristics of Gluten‐Free Bread

Gluten‐free bread products must meet sensory standards that satisfy consumer expectations, as taste and nutritional quality are primary factors influencing purchase intent (Stea and Pickering 2019). Additionally, aroma and color are among the most critical attributes affecting consumer buying decisions (Masih and Sharma 2016). In this literature review, organoleptic data from each study were compiled based on hedonic tests evaluating taste/flavor, color, and overall acceptability, using hedonic scales of 5, 7, 9, or 10 points, depending on the methodology employed in the respective (Table 1) studies (Table 2). For all scales, higher sensory evaluation scores indicate greater preference, with a score of 1 representing the least favorable rating.

**TABLE 2:** Oranoleptic characteristics of gluten‐free bread with addition of XG and HPMC.

Hydrocolloid	Type of flour/starch	Treatment	Taste/Flavor	Color	Overall acceptance	Scale	References
XG	Corn starch (80%) + zein (20%)	XG 2%	< 4*	—	±4*	9	Sadat et al. (2023)
XG	Hom Nil rice flour 100%	XG‐to‐CMC ratios 1:2	5.67 ± 1.67	—	6.10 ± 1.47	9	Chueamchaitrakun et al. (2016)
XG	Maize starch 100 g + mageu 456 g + sorghum meal fine 169 g + soybean flour 124 g	XG 0.5%	6.67 ± 1.97	Crust: 6.79 ± 1.79 Crumb: 7.18 ± 1.59	6.89 ± 1.61	9	McCarroll and de Kock (2017)
XG	Proso millet flour (100%)	XG 0.5%	±6*	Crust: < 7* Crumb: < 7*	> 6*	9	Tamilselvan et al. (2022)
XG	Rice flour (30%) + small broken riceberry flour (70%)	XG 1%	> 5*	< 7*	±6*	9	Rakkhumkaew et al. (2019)
XG	Rice flour (70%) + buckwheat (30%)	XG 2%	—	—	5.9 ± 0.6	9	Burešová et al. (2016)
XG	Rice flour + corn flour + quinoa flour (100%)	Quinoa flour 49% + laccase 2 U/g + XG 0.46%	6 ± 0.81	Crust: 7.33 ± 0.57	7.25 ± 0.5	9.0	Alizadeh‐Bahaabadi et al. (2022)
XG	Rice flour + potato flour + corn starch (100%)	Proportion of XG and G 0.75:0.25	3*	3*	< 3*	5	Sutrisno et al. (2021)
XG	Small broken rice flour 46.45 g	XG 1.0 g	5.40 ± 1.60	Crumb: 6.07 ± 1.28	5.73 ± 1.22	9	Numfon (2017)
XG	Small broken rice flour 46.45 g	XG 1.5 g	5.47 ± 1.36	Crumb: 5.67 ± 1.45	5.53 ± 1.60	9	
XG	Small broken rice flour 46.45 g	XG 2.0 g	5.87 ± 1.77	Crumb: 6.13 ± 1.36	6.13 ± 1.88	9	
HPMC	Corn starch (80%) + zein (20%)	HPMC 2%	±5*	—	> 5*	9	Sadat et al. (2023)
HPMC	Maize starch 100 g + mageu 456 g + sorghum meal fine 169 g + soybean flour 124 g	HPMC 0.8%	5.03 ± 1.76	Crust: 6.18 ± 1.99 Crumb: 5.82 ± 1.71	5.11 ± 1.67	9	McCarroll and de Kock (2017)
HPMC	Proso millet flour (100%)	HPMC 2.0%	< 7*	Crust color: ±8* Crumb color: < 8*	> 7*	9	Tamilselvan et al. (2022)
HPMC	Rice flour (50%) + millet flour (50%)	HPMC 1% + CRJ 3%	±5*	—	±5*	5	Abdollahzadeh et al. (2024)
HPMC	Rice flour (80%) + soy flour (20%)	HPMC 1.5%	4.46 ± 1.49	5.16 ± 1.17	—	7	Huerta et al. (2016)
HPMC	Rice starch 100%	WPI 0.37% + HPMC 4.35% + b‐glucan 1%	5.27 ± 1.08	7.10 ± 0.66	5.73 ± 1.26	9	Kittisuban et al. (2014)
HPMC	Sorghum flour (70%) + potato flour (30%)	WPC 15% + HPMC 3%	7.81	Crust: 7.57 Crumb: 7.38	7.52	9	Rustagi et al. (2018)
XG dan HPMC	Rice flour (52%) + potato starch (36%) + sweet cassava flour (12%)	PDCF 20.00% + HPMC 0.51% + XG 0.51%	6.56 ± 1.79	6.20 ± 1.80	6.80 ± 1.62	9	Ewerling et al. (2020)

*: Data are presented as mean +‐ sd, or +/‐ or < in accordance to results of the cited scientific articles.

Numfon (2017), reported that increasing the amount of XG (1.0–2.0 g) in gluten‐free bread made from small broken rice flour improved scores for taste (5.87 ± 1.77a), color (6.13 ± 1.36a), and overall acceptability (6.13 ± 1.88ab) compared to the control bread (3.33 ± 1.11b, 5.73 ± 1.10a, and 3.13 ± 1.19c, respectively). In a study by Lazaridou et al. (2007), the incorporation of CMC, pectin, xanthan gum, agarose, and β‐glucan enhanced the acceptability of gluten‐free bread relative to the control. Typically, the inclusion of hydrocolloids promotes starch granule cohesion, thereby contributing to desirable bread attributes such as proper structure, improved mouthfeel, and increased loaf volume.

In the study by McCarroll and de Kock (2017), gluten‐free bread formulated with HPMC received the lowest sensory acceptance score (5.11), which was significantly lower than that of breads containing other hydrocolloids (CMC, guar gum, and XG). In contrast, Sadat et al. (2023) evaluated the sensory acceptance of gluten‐free bread prepared with maize starch (80%) and zein (20%) and found that the sample containing 2% HPMC achieved the highest overall acceptance score compared to breads with guar gum, XG, lactic acid, or pregelatinized starch. The addition of HPMC significantly improved gluten‐free bread taste, with an average score of 5 (“neutral”) on a 9‐point hedonic scale. Moreover, HPMC‐treated bread scored higher in appearance, crust color, and crumb color than bread containing 0.5% XG. Panelists preferred the darker crust and whiter crumb observed in the 2% HPMC gluten‐free bread. Nevertheless, in this study, all evaluated gluten‐free bread samples achieved average scores above 5 across all sensory attributes.

The discrepancies between the results of McCarroll and de Kock (2017) and Sadat et al. (2023) may be attributed to differences in formulation and raw material composition. McCarroll and de Kock (2017) reported that, when using a blend of corn starch, mageu flour, fine meal sorghum, and soybean flour, gluten‐free bread formulated with XG achieved higher sensory scores than that with HPMC and other hydrocolloids. In contrast, Sadat et al. (2023) found that, in gluten‐free bread made from corn starch and zein, HPMC yielded higher sensory scores compared to XG and several other hydrocolloids. These contrasting results suggest that sensory acceptance is highly dependent on the type of flour or starch used as the primary ingredient. Nevertheless, in both studies, gluten‐free breads formulated with either XG or HPMC received average sensory scores above 5, which are considered acceptable (Lazaridou et al. 2007).

Tamilselvan et al. (2022) reported that the addition of 2% HPMC to proso millet gluten‐free bread resulted in a flavor score of 7 on a 9‐point hedonic scale. A similar positive effect of HPMC was observed by Tabrizi et al. (2023), in which increasing the concentration of HPMC (0.5%–1.5%) improved the overall acceptance score of gluten‐free bread formulated from quinoa, grass pea, and chestnut flours. In contrast, Burešová et al. (2016) found that the addition of 2% XG, without combination with other hydrocolloids, to gluten‐free bread made from rice and buckwheat flours yielded an overall acceptability score of 5.9 on a 9‐point hedonic scale. This value was lower than those obtained with other treatments (CMC, calcium caseinate, and sodium caseinate) in the same study. The XG‐treated gluten‐free bread was characterized by a dry, coarse crust and an excessively sticky crumb. The addition of xanthan resulted in the most pronounced effect on viscoelastic properties, yielding strengthened doughs compared to other hydrocolloids (Lazaridou et al. 2007).

Color is a critical visual attribute in food products, serving as an important determinant of consumer perception, preference, and purchase intent. In conventional wheat‐based bread, consumers typically favor a lighter crumb color, which is therefore frequently evaluated as one of the key indicators in sensory quality assessment (Franco et al. 2020; Huerta et al. 2016). However, the crust color is also a critical factor for acceptance, with golden‐brown surfaces being commonly perceived as indicative of optimal baking and desirable flavor (Mohammadi et al. 2014). In the context of gluten‐free bread, lower L* values generally indicate a darker crust (Ziobro et al. 2013). Each of these parameters—L*, a*, and b*—influences the perceived color of gluten‐free bread in distinct ways, which makes it challenging to develop formulations that match the visual characteristics of wheat bread. Nevertheless, many studies emphasize the L* value as a primary indicator of bread color quality (Gallagher et al. 2003; Nunes et al. 2009; Sabanis et al. 2009a). Table 3 demonstrates the color attributes of gluten‐free bread with incorporated XG and HPMC.

**TABLE 3:** Color characteristics of gluten free bread with addition of XG and HPMC.

Hydrocolloid	Type of flour/starch	Treatment	Crumb			Crust			References
			L*	a*	b*	L*	a*	b*	
XG	Corn starch 60 g	MF CGM 20 g + XG 5 g	85.94 ± 0.40	5.83 ± 0.61	76.70 ± 1.12	—	—	—	Ozturk and Mert (2018)
XG	Corn starch 70 g	MF CGM 20 g + XG 5 g	89.59 ± 0.21	1.87 ± 0.32	62.46 ± 0.52	—	—	—	
XG	Corn starch 80 g	MF CGM 20 g + XG 5 g	91.48 ± 0.17	0.70 ± 0.34	54.61 ± 0.60	—	—	—	
XG	Kapac rice flour (94%) + cassava starch (4%)	XG to GG mass ratio of 0.71	—	—	—	24.53 ± 0.78	17.72 ± 0.25	25.51 ± 0.71	Cajas Locke et al. (2019)
XG	Light buckwheat flour 88.2 g + chia flour 9.8 g	XG 2 g	50.62 ± 0.47	4.21 ± 0.002	12.84 ± 0.12	51.75 ± 1.07	10.01 ± 0.46	24.86 ± 0.32	Coronel et al. (2021)
XG	Maize starch (100%)	XG 2%	—	—	—	71.26 ± 1.92	0.06 ± 0.09	9.72 ± 1.93	Belorio and Gómez (2020)
XG	Proso millet flour (100%)	XG 0.5%	62.02 ± 0.35	2.65 ± 0.03	19.93 ± 0.13	55.70 ± 0.38	5.99 ± 0.08	19.36 ± 0.12	Tamilselvan et al. (2022)
XG	Proso millet flour (100%)	XG 1.0%	65.06 ± 0.16	2.52 ± 0.06	20.27 ± 0.05	56.29 ± 0.39	6.17 ± 0.02	19.37 ± 0.11	
XG	Proso millet flour (100%)	XG 1.5%	66.18 ± 0.21	2.42 ± 0.17	20.24 ± 0.12	55.15 ± 0.16	6.62 ± 0.10	19.01 ± 0.14	
XG	Proso millet flour (100%)	XG 2.0%	66.43 ± 0.29	2.32 ± 0.05	19.99 ± 0.04	54.05 ± 0.57	4.63 ± 0.04	17.77 ± 0.23	
XG	Rice flour (100%)	XG 2%	61.75 ± 4.68	1.92 ± 3.48	7.67 ± 2.53	37.26 ± 3.72	17.08 ± 2.10	17.24 ± 2.22	Franco et al. (2020)
XG	Rice flour (100%)	XG 1.5% + CMC 0.5%	65.68 ± 7.46	2.53 ± 3.32	9.24 ± 2.22	43.15 ± 2.75	18.48 ± 0.49	19.05 ± 1.02	
XG	Rice flour (100%)	XG 1% + CMC 1%	67.76 ± 3.38	0.91 ± 2.36	7.83 ± 5.52	51.30 ± 3.15	19.28 ± 1.76	26.62 ± 2.01	
XG	Rice flour (100%)	XG 0.5% + CMC 1.5%	67.81 ± 2.27	0.95 ± 0.27	7.61 ± 0.69	47.20 ± 8.01	18.76 ± 1.74	23.35 ± 4.30	
XG	Rice flour (100%)	XG 4%	73.0 ± 0.0	0.2 ± 0.0	14.0 ± 0.3	—	—	—	Jang et al. (2018)
XG	Rice flour (100%)	Rosellle seed powder 30% + egg white powder 25% + XG 0.73%	—	—	—	61.06 ± 0.50	2.83 ± 0.02	11.27 ± 0.80	Zarringhalami et al. (2021)
XG	Rice flour (100%)	XG 0.25%	> 70*	> 10*	> 35*	—	—	—	Fujii et al. (2023)
XG	Rice flour (100%)	XG 0.50%	> 70*	±10*	±35*	—	—	—	
XG	Rice flour (100%)	XG 0.75%	> 70*	< 20*	> 40*	—	—	—	
XG	Rice flour (100%)	XG 1.0%	> 70*	±20*	> 40*	—	—	—	
XG	Rice flour (100%)	XG 2%	—	—	—	75.05 ± 0.83	4.48 ± 0.08	20.25 ± 1.22	Belorio and Gómez (2020)
XG	Rice flour (30%) + small broken riceberry flour (70%)	XG 1%	26.45 ± 0.14	8.60 ± 0.08	3.62 ± 0.15	—	—	—	Rakkhumkaew et al. (2019)
XG	Rice flour (95%) + DHF (5%)	XG 0.25%	57.81 ± 3.54	1.57 ± 0.26	11.22 ± 0.99	47.96 ± 3.86	6.96 ± 0.97	18.61 ± 1.76	Tunç and Kahyaoglu (2016)
XG	Rice flour + corn flour + corn starch (100%)	XG 1%	86.86	−17.37	35.12	85.81	−15.42	38.6	Naji‐Tabasi and Mohebbi (2015)
XG	Rice flour + corn flour + quinoa flour (100%)	Quinoa flour 49% + laccase 2 U/g + XG 0.46%	—	—	—	49.78 ± 0.07	8.80 ± 0.09	26.44 ± 0.25	Alizadeh‐Bahaabadi et al. (2022)
XG	Rice flour 45 g + cassava starch 45 g + soy flour 10 g	Gl 0.25% + XG 0.25%	—	—	—	76.1 ± 0.8	4.4 ± 0.8	26.9 ± 1.8	Sciarini et al. (2023)
XG	Rice flour 45 g + cassava starch 45 g + soy flour 10 g	Gl 0.63% + XG 0.63%	—	—	—	76.0 ± 1.1	3.0 ± 0.7	22.4 ± 1.4	
XG	Rice flour 45 g + cassava starch 45 g + soy flour 10 g	GG 0.25% + XG 0.25%	—	—	—	80.5 ± 0.5	2.4 ± 0.4	22.5 ± 1.3	
XG	Rice flour 45 g + cassava starch 45 g + soy flour 10 g	GG 0.63% + XG 0.63%	—	—	—	80.3 ± 0.9	1.5 ± 0.2	15.6 ± 1.2	
HPMC	Colocasia cormles rhizome flour (100%)	HPMC 2%	56.87 ± 0.66	7.09 ± 0.82	23.97 ± 0.66	—	—	—	Calle et al. (2020)
HPMC	Maize starch (100%)	HPMC 2%	—	—	—	82.09 ± 0.04	2.64 ± 0.14	19.32 ± 0.22	Belorio and Gómez (2020)
HPMC	Maize starch (30%) + yellow maize flour (70%)	HPMC 4% + SBF 3%	72.38	−3.19	28.96	65.44	5.82	35.2	Djordjević et al. (2019)
HPMC	Maize starch (30%) + yellow maize flour (70%)	HPMC 4% + AF 3%	70.51	0.46	21.54	55.88	10.86	30.8	
HPMC	Maize starch (30%) + yellow maize flour (70%)	HPMC 4% + SBF 7%	72.68	−3.59	20.7	56.5	9.29	30.83	
HPMC	Maize starch (30%) + yellow maize flour (70%)	HPMC 4% + AF 7%	61.37	3.27	21.93	53.15	12.19	27.7	
HPMC	Proso millet flour (100%)	HPMC 2.0%	60.82 ± 0.21	2.19 ± 0.02	19.15 ± 0.10	35.41 ± 0.47	12.83 ± 0.14	15.04 ± 0.17	Tamilselvan et al. (2022)
HPMC	Quinoa flour (27/69%) + grass pea flour (30%) + chestnut flour (42/31%)	HPMC 0.5%	72.13 ± 0.03	10.38 ± 0.07	17.11 ± 0.1	60.02 ± 0.05	20.47 ± 0.085	20.33 ± 0.03	Tabrizi et al. (2023)
HPMC	Quinoa flour (27/69%) + grass pea flour (30%) + chestnut flour (42/31%)	HPMC 1.0%	73.00 ± 0.10	10.66 ± 0.02	17.49 ± 0.04	60.16 ± 0.01	20.53 ± 0.01	19.81 ± 0.11	
HPMC	Quinoa flour (27/69%) + grass pea flour (30%) + chestnut flour (42/31%)	HPMC 1.5%	73.21 ± 0.02	11.01 ± 0.04	18.10 ± 0.04	60.03 ± 0.02	20.47 ± 0.03	21.03 ± 0.03	
HPMC	Rice flour (100%)	SFE 0.7% + NE 1.9% + BBG 2.95% + Water 100%	79	—	—	67	—	—	Pérez‐Quirce et al. (2014)
HPMC	Rice flour (100%)	HPMC 4%	85.4 ± 0.8	0.1 ± 0.1	11.3 ± 0.8	—	—	—	Jang et al. (2018)
HPMC	Rice flour (100%)	HPMC 2%	—	—	—	81.68 ± 3.05	1.64 ± 0.25	17.15 ± 0.68	Belorio and Gómez (2020)
HPMC	Rice flour (50%) + millet flour (50%)	HPMC 1% + CRJ 3%	—	—	—	42.82 ± 0.1	3.06 ± 0.08	14.20 ± 0.1	Abdollahzadeh et al. (2024)
HPMC	Rice flour (50%) + millet flour (50%)	HPMC 1% + CRJ 4%	—	—	—	35.24 ± 0.25	3.32 ± 0.23	14.03 ± 0.05	
HPMC	Rice flour (66,66%) + wholemeal buckwheat flour (33,33%)	Water 85% + HPMC 1%	64 ± 1	2.3 ± 0.3	12 ± 1	47.8 ± 0.5	9.0 ± 0.4	29.1 ± 0.1	Baldino et al. (2018)
HPMC	Rice flour (66,66%) + wholemeal buckwheat flour (33,33%)	Water 100% + HPMC 1%	63 ± 1	1.9 ± 0.1	10 ± 2	52.3 ± 0.3	8.9 ± 0.2	31.6 ± 0.8	
HPMC	Rice flour 38 g + corn starch 38 g	WOP 1.5% + HPMC 1.5%	77.59 ± 2.27	−2.91 ± 0.43	13.96 ± 1.38	72.09 ± 4.59	2.17 ± 1.45	24.14 ± 2.54	Tufaro et al. (2022)
HPMC	Rice flour 38 g + corn starch 38 g	FOP 1.5% + HPMC 1.5%	76.47 ± 1.90	−3.01 ± 0.23	13.23 ± 1.06	68.80 ± 2.91	2.79 ± 0.86	25.23 ± 2.15	
HPMC	Rice starch (100%)	WPI 0.37% + HPMC 4.35% + b‐glucan 1%	79.83 ± 0.08	—	—	—	—	—	Kittisuban et al. (2014)
HPMC	Sorghum flour (70%) + potato flour (30%)	WPC 15% + HPMC 3%	64.68	7.77	21.29	52.3	15.21	31.59	Rustagi et al. (2018)

*: Data are presented as mean +‐ sd, or +/‐ or < in accordance to results of the cited scientific articles.

Tamilselvan et al. (2022) reported that incorporating XG (0.5%–2.0%) into proso millet flour gluten‐free bread increased the L* value of the crumb, indicating a lighter color. Similar trends were observed by Jang et al. (2018) and Fujii et al. (2023). On the other hand, the a* value of the crumb decreased with higher XG concentrations. This reduction in a* was attributed to the decrease in specific volume caused by XG, leading to fewer air cells. In addition, elevated XG levels were suggested to inhibit the Maillard reaction between rice flour proteins and added sugars during baking, likely due to increased dough viscosity.

By contrast, Franco et al. (2020) reported different results. In their study on 100% rice flour gluten‐free bread, the addition of 2% XG resulted in reduced L* and b* values for both the crumb and crust compared to the control. Interestingly, the a* and b* values of the crust suggested a more golden coloration relative to the control. Among the tested formulations, the treatment with 2% XG alone yielded the darkest crumb, which the authors attributed to the higher sugar content and inclusion of egg white in the recipe. Added sugars are known to promote caramelization and Maillard reactions, thereby contributing to darkening color (Sabanis et al. 2008).

Zarringhalami et al. (2021) linked changes in the characteristics of gluten‐free bread made from rice flour and XG to the addition of roselle seed powder and egg white powder, which produced a darker crust. The optimized formulation exhibited higher redness (a*), likely due to the naturally dark hue of roselle seed powder. Protein enrichment also promoted the Maillard reaction, consistent with previous findings (Aguilar et al. 2015; Krupa‐Kozak et al. 2013; Pico et al. 2019). Overall, gluten‐free bread color characteristics are strongly influenced by flour type (Pomeranz 1960). The crumb color typically reflects the ingredient color, as it does not reach the high surface temperatures necessary for Maillard browning or caramelization (Sabanis et al. 2009b). Even when the same HPMC concentration is used, different flour types can yield varied color outcomes.

Jang et al. (2018) reported that rice flour gluten‐free bread samples with HPMC (4%) had a higher crumb L* value (85.4 ± 0.8) compared to that of the same level of XG (73.0 ± 0.0). They attributed this to the greater number of air cells in the HPMC gluten‐free bread. In contrast, the control samples without hydrocolloid showed a lower L* value, indicating fewer air cells and higher density. These findings are consistent with Belorio and Gómez (2020), who found that gluten‐free bread made with XG appeared darker (lower L* value) than gluten‐free bread made with HPMC. Such differences in hydrocolloid type can influence water activity, thereby affecting the extent of the Maillard reaction (Pereyra Gonzales et al. 2010). Moreover, water addition levels also play a role in gluten‐free bread color, as higher water content can dilute reaction precursors and reduce Maillard reaction rates (Baldino et al. 2018; Pérez‐Quirce et al. 2014).

Taken together, current evidence indicates that crust darkening in gluten‐free bread is primarily the result of Maillard reactions between starch‐derived sugars and proteins, while hydrocolloids such as XG and HPMC influence the extent of these reactions indirectly through changes in dough viscosity, water activity, and crumb porosity. Thus, color outcomes in gluten‐free bread should be understood as the combined effect of formulation components and hydrocolloid–matrix interactions rather than the action of hydrocolloids alone.

### 3.3 The Role of XG and HPMC in the Textural Characteristics of Gluten‐Free Bread

Vidaurre‐Ruiz et al. (2019) compared the texture characteristics of gluten‐free bread made from potato starch and corn starch with the addition of 0.5% XG. Gluten‐free bread formulated with potato starch and XG showed lower hardness values (1.90 ± 0.00a) than gluten‐free bread made with corn starch (2.42 ± 0.04b), indicating that the interaction between XG and potato starch produces a softer crumb. Similar findings were reported by Horstmann et al. (2018), who observed that gluten‐free bread made from potato starch and XG was softer compared to gluten‐free bread prepared with potato starch and locust bean gum. The lower hardness values observed in breads formulated with xanthan gum are attributed to its negatively charged molecules, which generate repulsive forces that limit starch granule swelling and restrict amylose leaching. Consequently, the reduced availability of leached amylose lowers the extent of amylose retrogradation, resulting in a softer crumb texture (Horstmann et al. 2018).

The use of xanthan gum (XG) and hydroxypropyl methylcellulose (HPMC) in gluten‐free bread formulations has been shown to markedly influence textural attributes (Table 4). Ozturk and Mert (2018) reported that incorporating 1% XG into corn starch‐based gluten‐free bread reduced hardness from 2.85 ± 0.10 to 2.12 ± 0.08 N and increased cohesiveness and springiness by approximately 12% and 9%, respectively, compared with the control (without XG). Similarly, Chueamchaitrakun et al. (2016) observed that the addition of XG (0.5%–1.5%) to Hom Nil rice flour (HNRF)‐based gluten‐free bread significantly decreased hardness (from 3.10 ± 0.14 to 2.36 ± 0.11 N), chewiness, and springiness values (*p* < 0.05).

**TABLE 4:** Textural characteristics of gluten‐free bread with the addition of XG and HPMC.

Hydrocolloid	Type of flour/starch	Treatment	Hardness	Cohesiveness	Springiness	Chewiness	References
XG	Corn starch (100%)	XG 0.5%	2.37 ± 0.25 N	0.01 ± 0.00	0.01 ± 0.00	1.37 ± 0.12	Vidaurre‐Ruiz et al. (2019)
XG	Corn starch (80%) + zein (20%)	XG 2%	3115 ± 270 N	0.38 ± 0.03	—	—	Sadat et al. (2023)
XG	Corn starch 60 g	MF CGM 20 g + 5 g XG	< 2.5 N*	> 0.8*	< 6 s*	—	Ozturk and Mert (2018)
XG	Corn starch 70 g	MF CGM 20 g + 5 g XG	±2.5 N*	< 0.8*	±5.5 s*	—	
XG	Corn starch 80 g	MF CGM 20 g + 5 g XG	< 5 N	> 0.6*	> 5 s*	—	
XG	Hom Nil rice flour 100%	XG‐to‐CMC ratios 1:2	7.02 ± 1.94 g	—	0.73 ± 0.08 mm	1.81 ± 1.39 gmm	Chueamchaitrakun et al. (2016)
XG	Kapac rice flour (94%) + cassava starch (4%)	XG to GG mass ratio of 0.71	20.96 ± 0.85 N	0.29 ± 0.01	—	4.89 ± 0.24	Cajas Locke et al. (2019)
XG	Light buckwheat flour 88.2 g + chia flour 9.8 g	XG 2 g	8.54 ± 0.29 N	0.50 ± 0.01	0.83 ± 0.01 mm	3.50 ± 0.24 Nxmm	Coronel et al. (2021)
XG	Maize starch (100%)	XG 2%	19.58 ± 1.55 N	0.606 ± 0.037	0.964 ± 0.002	—	Belorio and Gómez (2020)
XG	Potato starch (100%)	XG 0.5%	1.90 ± 0.00 N	0.04 ± 0.00	0.01 ± 0.00	7.38 ± 0.32	Vidaurre‐Ruiz et al. (2019)
XG	Proso millet flour (100%)	XG 0.5%	117.19 ± 11.55 N	—	0.46 ± 0.02 mm	8.16 ± 0.63 N	Tamilselvan et al. (2022)
XG	Proso millet flour (100%)	XG 1.0%	135.06 ± 4.71 N	—	0.45 ± 0.02 mm	11.28 ± 0.63 N	
XG	Proso millet flour (100%)	XG 1.5%	140.50 ± 12.66 N	—	0.43 ± 0.02 mm	12.62 ± 0.84 N	
XG	Proso millet flour (100%)	XG 2.0%	139.31 ± 10.24 N	—	0.45 ± 0.01 mm	14.72 ± 1.09 N	
XG	Rice flour (100%)	XG 2%	110.72 ± 17.84 gf	0.75 ± 0.02	—	66.07 ± 19.17 gf. mm	Franco et al. (2020)
XG	Rice flour (100%)	XG 1% + CMC 1%	106.54 ± 52.24 gf	0.72 ± 0.02	—	48.11 ± 20.12 gf. mm	
XG	Rice flour (100%)	XG 0.5% + CMC 1.5%	417.00 ± 19.51 gf	0.75 ± 0.02	—	262.51 ± 31.58 gf. mm	
XG	Rice flour (100%)	XG 1.5% + CMC 0.5%	949.76 ± 248.59 gf	0.71 ± 0.00	—	600.70 ± 178.90 gf. mm	
XG	Rice flour (100%)	DATEM 0.5% + XG 0.5%*	0.58 N	0.5	91.2%	0.26 N	Demirkesen et al. (2014)
XG	Rice flour (100%)	DATEM 0.5% + XG‐LBG 0.5%*	0.38 N	0.55	94.3%	0.20 N	
XG	Rice flour (100%)	DATEM 0.5% + XG‐G 0.5%*	0.35 N	0.56	96.7%	0.19 N	
XG	Rice flour (100%)	XG 2%	9.04 ± 3.00 N	0.807 ± 0.024	0.922 ± 0.043	—	Belorio and Gómez (2020)
XG	Rice flour (50%) + maize flour (30%) + quinoa flour (20%)	XG 1.5%	3574 g	0.453	0.864	1353 g	Encina‐Zelada et al. (2018)
XG	Rice flour (50%) + maize flour (30%) + quinoa flour (20%)	XG 2.5%	3355 g	0.513	0.877	1407 g	
XG	Rice flour (70%) + buckwheat (30%)	XG 2%	9.1 ± 0.3 N	614 ± 10 (10^‐3)	—	116 ± 3 N s	Burešová et al. (2016)
XG	Rice flour (95%) + DHF (5%)	XG 0.25%	1087.61 ± 271.23 g	0.679% ± 0.02%	0.622 ± 0.05 mm	455.45 ± 104.93 gmm	Tunç and Kahyaoglu (2016)
XG	Rice flour + corn flour + quinoa flour (100%)	Quinoa flour 49% + laccase 2 U/g + XG 0.46%	1913.49 ± 6.08 gf	0.52 ± 0.009	6.52 ± 0.04 mm	—	Alizadeh‐Bahaabadi et al. (2022)
XG	Rice flour + potato flour + corn starch (100%)	Proportion of XG and G 0.75:0.25	—	0.920 ± 0.017	3.973 ± 0.0985 mm	—	Sutrisno et al. (2021)
XG	Rice flour 45 g + cassava starch 45 g + soy flour 10 g	Gl 0.25% + XG 0.25%	—	—	—	5.4 ± 1.4	Sciarini et al. (2023)
XG	Rice flour 45 g + cassava starch 45 g + soy flour 10 g	Gl 0.63% + XG 0.63%	—	—	—	6.3 ± 0.4	
XG	Rice flour 45 g + cassava starch 45 g + soy flour 10 g	GG 0.25% + XG 0.25%	—	—	—	5.0 ± 0.8	
XG	Rice flour 45 g + cassava starch 45 g + soy flour 10 g	GG 0.63% + XG 0.63%	—	—	—	5.8 ± 0.8	
XG	Small broken rice flour 46.45 g	XG 1.0 g	±8.0 N*	—	> 6.0 N*	—	Numfon (2017)
XG	Small broken rice flour 46.45 g	XG 1.5 g	< 8.0 N*	—	> 5.0 N*	—	
XG	Small broken rice flour 46.45 g	XG 2.0 g	< 6.0 N*	—	±5.0 N*	—	
XG	Tapioca starch (60%) + millet flour (40%)	XG 1%	±3.5 N*	0.3*	0.4*	—	Chakraborty et al. (2020)
XG	Tapioca starch (60%) + millet flour (40%)	XG 1.5%	±3.4 N	0.4*	0.4*	—	
XG	Tapioca starch (60%) + millet flour (40%)	XG 2.0%	±3.3 N*	0.5*	0.5*	—	
XG	Tapioca starch (60%) + millet flour (40%)	XG 2.5%	±3.3 N*	0.5*	0.5*	—	
HPMC	Colocasia cormles rhizome flour (100%)	HPMC 2%	316 ± 12 g	0.348 ± 0.028	0.441 ± 0.087	50 ± 4 g	Calle et al. (2020)
HPMC	Corn starch (80%) + zein (20%)	HPMC 2%	949 ± 52 N	0.38 ± 0.06	—	—	Sadat et al. (2023)
HPMC	Maize starch (100%)	HPMC 2%	1.44 ± 0.12 N	0.754 ± 0.030	1.011 ± 0.023	—	Belorio and Gómez (2020)
HPMC	Maize starch (30%) + yellow maize flour (70%)	HPMC 4% + SBF 3%	2.29 N	—	—	100.23	Djordjević et al. (2019)
HPMC	Maize starch (30%) + yellow maize flour (70%)	HPMC 4% + AF 3%	2.60 N	—	—	91.06	
HPMC	Maize starch (30%) + yellow maize flour (70%)	HPMC 4% + SBF 7%	3.30 N	—	—	142.04	
HPMC	Maize starch (30%) + yellow maize flour (70%)	HPMC 4% + AF 7%	2.10 N	—	—	78.13	
HPMC	Proso millet flour (100%)	HPMC 2.0%	94.70 ± 4.47 N	—	0.31 ± 0.02 mm	2.40 ± 0.45 N	Tamilselvan et al. (2022)
HPMC	Rice flour (100%)	HPMC 4% + WPC 4%	5.86 ± 0.73 N	0.68 ± 0.01	1.13 ± 0.19 (J/F)	4.52 ± 0.98	Srikanlaya et al. (2018)
HPMC	Rice flour (100%)	SFE 0.7% + NE 1.9% + BBG 2.95% + Water 100%	—	0.56	0.7	0.6	Pérez‐Quirce et al. (2014)
HPMC	Rice flour (100%)	HPMC 2%	79 ± 5 g	—	78.20% ± 1.71%	—	Zhao et al. (2021)
HPMC	Rice flour (100%)	HPMC 2% + PGA 0.5%	98 ± 2 g	—	92.40% ± 3.71%	—	
HPMC	Rice flour (100%)	HPMC 2% + PGA 1.0%	100 ± 4 g	—	97.58% ± 5.70%	—	
HPMC	Rice flour (100%)	HPMC 1% + PGA 0.5%	527 ± 14 g	—	92.57% ± 0.90%	—	
HPMC	Rice flour (100%)	HPMC 1.5% + PGA 0.5%	101 ± 4 g	—	92.64% ± 1.45%	—	
HPMC	Rice flour (100%)	DATEM 0.5% + HPMC 0.5%*	0.40 N	0.56	95.4%	0.21 N	Demirkesen et al. (2014)
HPMC	Rice flour (100%)	HPMC 0.5%	2.18 ± 0.43 × 10^3^ Pa	0.75 ± 0.01	—	—	Imamura et al. (2021)
HPMC	Rice flour (100%)	HPMC 2%	42.44 ± 0.21 N	0.656 ± 0.023	0.796 ± 0.004	—	Belorio and Gómez (2020)
HPMC	Rice flour 38 g + corn starch 38 g	WOP 1.5% + HPMC 1.5%	0.315 ± 0.032 N	—	—	—	Tufaro et al. (2022)
HPMC	Rice flour 38 g + corn starch 38 g	FOP 1.5% + HPMC 1.5%	0.284 ± 0.027 N	—	—	—	
HPMC	Rice starch (100%)	WPI 0.37% + HPMC 4.35% + b‐glucan 1%	78.47 ± 1.81 g	0.53 ± 0.03	—	41.63 ± 2.29	Kittisuban et al. (2014)
HPMC	Sorghum flour (70%) + potato flour (30%)	WPC 15% + HPMC 3%	4228.2 g	—	—	—	Rustagi et al. (2018)
HPMC	Unripe banana flour (100%)	Water 52% + UBF‐P 28% + HPMC 6%	2.90 ± 1.20 N	—	—	—	Hernández‐Aguirre et al. (2019)
XG dan HPMC	Colocasia cormles rhizome flour (100%)	HPMC 0.29% + XG 0.21% + GG 0.50%	323 ± 23 g	0.313 ± 0.044	0.434 ± 0.135	45 ± 6 g	Calle et al. (2020)
XG dan HPMC	Rice flour (50%) + maize flour (30%) + quinoa flour (20%)	XG 0.24% + GG 0.60% + HPMC 3.16%	2082 g				Encina‐Zelada et al. (2019)

*: Data are presented as mean +‐ sd, or +/‐ or < in accordance to results of the cited scientific articles.

These reductions in hardness and chewiness are attributed to the ability of XG to enhance dough viscosity, stabilize air bubbles during fermentation, and improve gas retention in the matrix [59]. Additionally, XG's high water‐binding capacity increases starch hydration and limits retrogradation during baking, leading to a softer crumb and improved elasticity (Chueamchaitrakun et al. 2016). The resulting network is more cohesive and flexible, contributing to the improved texture profile of gluten‐free bread.

Generally, the addition of hydrocolloids tends to reduce gluten‐free bread hardness (Culetu et al. 2021). However, contrasting results were reported by Coronel et al. (2021), where the incorporation of chia flour (9.8 g) together with XG (2 g) into light buckwheat flour (88.2 g) significantly increased gluten‐free bread hardness. This was attributed to the lower specific volume of the gluten‐free bread formulated with XG and chia flour. A negative correlation between specific volume and hardness value has also been observed in previous studies Belorio and Gómez (2020). The combination of XG and chia flour also increased chewiness, even exceeding that of the control samples, which may be related to the higher hardness value. Furthermore, the cohesiveness value significantly increased (0.50 ± 0.01) when both XG and chia flour were added. The addition of fiber‐rich ingredients, such as chia flour, has been reported to reduce bread texture quality (Katina et al. 2006).

Meanwhile, Tamilselvan et al. (2022) reported that the addition of HPMC and XG significantly reduced the hardness of proso millet flour gluten‐free bread. Similarly, Sabanis and Tzia (2011) observed a decrease in crumb hardness with the incorporation of HPMC, attributing this effect to the formation of hydrogen bonds between starch and hydrocolloids, which can slow the rate of starch retrogradation. The addition of XG increased crumb springiness and chewiness, whereas gluten‐free bread containing HPMC exhibited lower values (*p* < 0.05). In formulations with HPMC, the relative reduction in hardness was greater, which in turn significantly influenced the chewiness value.

Belorio and Gómez (2020) reported that gluten‐free bread formulated with HPMC exhibited substantially lower hardness than that made with XG, likely due to the higher specific volume of HPMC samples. In general, greater specific volume is associated with lower hardness, a relationship also supported by previous studies on HPMC (Sabanis and Tzia 2011) and other research linking specific volume with hardness (Mancebo et al. 2017; Martínez and Gómez 2017). Rice flour gluten‐free bread containing XG demonstrated higher springiness, cohesiveness, and resilience compared to that made with HPMC.

Generally, the addition of hydrocolloids reduces bread hardness. However, their effect also depends on the type of flour used (Sasaki 2018). Gluten‐free bread crumb texture is influenced by the ingredients and formulation, while factors such as hydrocolloid type, concentration, and interactions significantly affect crumb hardness (Horstmann et al. 2018).

A study by Rustagi et al. (2018) investigated gluten‐free bread made from sorghum flour and potato flour, combining 3% HPMC with 15% whey protein concentrate (WPC). The control samples (without HPMC) exhibited texture defects, such as a cracked surface, due to the absence of HPMC as an emulsifying agent, which made the dough more susceptible to separation during proofing. Gluten‐free bread hardness ranged from 4228.2 g (HPMC + WPC) to 5504.89 g (control), with the reduction in hardness attributed to the texture‐enhancing effects of HPMC and WPC.

Different gluten‐free bread‐making processes can produce varying textures, with water content being a key factor. Encina‐Zelada et al. (2019) reported that a higher xanthan gum level (3.5%) at a constant water level (90%) increased crumb hardness in gluten‐free bread made from 50% rice flour, 30% corn flour, and 20% quinoa flour. Increasing water content decreased hardness, producing gluten‐free bread with greater specific volume and a softer crumb. In addition to water content, combining hydrocolloids with emulsifiers such as DATEM has been shown to reduce hardness and improve product quality in terms of specific volume and sensory properties (Demirkesen et al. 2010).

HPMC reduces water diffusion and loss in gluten‐free bread while limiting interactions between starch and protein macromolecules, resulting in a softer crumb and a slower staling rate during storage (Tufaro et al. 2022). Similarly, XG exhibits water‐retention properties, reducing water evaporation during baking. The slower staling rate is associated with a reduced rate of amylopectin recrystallization during storage (Liu et al. 2018). Furthermore, the decreased hardness in gluten‐free bread containing XG is partly attributed to the negative charges on XG molecules. The acetate and pyruvate groups in XG facilitate higher water absorption through hydrogen bonding, leading to a softer texture (Encina‐Zelada et al. 2018).

### 3.4 The Role of XG and HPMC in the Nutritional Characteristics of Gluten‐Free Bread

Gluten‐free bread often has low nutritional quality because it is typically made from starch and lacks fiber, vitamins, and other essential nutrients (Mariani et al. 1998). This poses a challenge in the development of gluten‐free products, particularly in meeting the nutritional needs of individuals with celiac disease. To address this issue, several studies have incorporated specific ingredients aimed at improving the nutritional profile of gluten‐free bread (Ewerling et al. 2020; Rustagi et al. 2018) (Table 5).

**TABLE 5:** Nutritional characteristics of gluten‐free bread with addition of XG and HPMC.

Hydrocolloid	Type of flour/starch	Treatment	Ash (g/100 g)	Fiber/starch (g/100 g)	Fat content (g/100 g)	Protein (g/100 g)	Carbo‐hydrates (g/100 g)	References
XG	Hom Nil rice flour (100%)	XG‐to‐CMC ratios 1:2	2.12%	Crude fiber: 1.36%	6.88%	6.74%	54.10%	Chueamchaitrakun et al. (2016)
XG	Light Buckwheat Flour 88.2 g + chia flour 9.8 g	XG 2 g	2.13 ± 0.01	Crude fiber: 1.28 ± 0.004	Oil: 1.08 ± 0.04	9.02 ± 0.02	86.49 ± 0.01	Coronel et al. (2021)
XG	Rice flour (100%)	Rosellle seed powder 30% + egg white powder 25% + XG 0.73%	0.81 ± 0.02	Crude fiber: 5.35 ± 0.01	Oil: 3.23 ± 0.12	11.98 ± 0.02	—	Zarringhalami et al. (2021)
XG	Rice flour (30%) + small broken riceberry flour (70%)	XG 1%	0.79	—	4.27	4.04	46	Rakkhumkaew et al. (2019)
XG	Rice starch (10%) + sorghum flour (90%)	XG 3%	—	Total dietary fiber: 12.0 Soluble dietary fiber: 3.3 Insoluble dietary fiber: 8.7	—	7.4	—	Ari Akin et al. (2019)
HPMC	Colocasia cormles rhizome flour (100%)	HPMC 2%	—	Rapid digestible starch: 4.38 ± 0.40 Slowly digestible starch: 6.895 ± 1.309 Resistance starch: 6.730 ± 0.393	—	—	—	Fujii et al. (2023)
HPMC	Maize starch (30%) + yellow maize flour (70%)	HPMC 4% + SBF 3%	2.60 ± 0.017	Total dietary fiber: 4.98 ± 0.024	Lipid: 2.31 ± 0.169	2.14 ± 0.131	36.10 ± 0.617	Djordjević et al. (2019)
HPMC	Maize starch (30%) + yellow maize flour (70%)	HPMC 4% + AF 3%	2.55 ± 0.004	4.56 ± 0.200	2.86 ± 0.056	2.80 ± 0.013	42.66 ± 0.494	
HPMC	Maize starch (30%) + yellow maize flour (70%)	HPMC 4% + SBF 7%	2.58 ± 0.004	6.07 ± 0.078	2.46 ± 0.135	2.14 ± 0.045	35.51 ± 0.774	
HPMC	Maize starch (30%) + yellow maize flour (70%)	HPMC 4% + AF 7%	2.64 ± 0.002	5.89 ± 0.100	2.93 ± 0.051	2.98 ± 0.074	41.26 ± 0.867	
HPMC	Sorghum flour (70%) + potato flour (30%)	WPC 15% + HPMC 3%	Acid insoluble ash 0.134	Crude fiber 2.80	8.97	10.48	—	Rustagi et al. (2018)
HPMC	Tapioca starch (10%) + sorghum flour (90%)	HPMC 3%	—	Total dietary fiber: 5.1 Soluble dietary fiber: 0.3 Insoluble dietary fiber: 4.8	—	7.1	—	Ari Akin et al. (2019)
HPMC	Unripe banana flour (100%)	Water 52% + UBF‐P 28% + HPMC 6%	—	3.49 ± 0.30	—	—	—	Hernández‐Aguirre et al. (2019)
XG and HPMC	Colocasia cormles rhizome flour (100%)	HPMC 0.29% + XG 0.21% + GG 0.50%	—	Rapid digestible starch: 7.01 ± 0.40 Slowly digestible starch: 5.809 ± 0.094 Resistance starch: 6.492 ± 0.362	—	—	—	Calle et al. (2020)
XG and HPMC	Rice flour (52%) + potato starch (36%) + sweet cassava flour (12%)	PDCF 20.00% + HPMC 0.51% + XG 0.51%	2.38 ± 0.05		Total lipids: 13.06 ± 0.33	Crude protein: 12.00 ± 0.62	72.55 ± 0.86	Ewerling et al. (2020)

The nutritional value of gluten‐free bread can be enhanced by incorporating fiber or protein‐rich ingredients. For example, Djordjević et al. (2019) enriched gluten‐free bread with fiber, while Rustagi et al. (2018) incorporated whey protein concentrate (WPC) into gluten‐free bread made from sorghum and potato flour. The optimized formulation showed higher protein content than the control samples, which the authors attributed to the addition of WPC.

Hydrocolloids, such as XG and HPMC, primarily enhance texture, volume, and dough stability rather than nutritional value. Lazaridou et al. (2007) reported that hydrocolloid addition enhances the physical properties of gluten‐free bread without significantly affecting its nutrient profile. Similarly, Sciarini et al. (2023) highlighted that hydrocolloids act at a physicochemical level, interacting with starch and protein to strengthen the dough network, but do not contribute macronutrients like protein or fiber. Consequently, their role is largely confined to enhancing sensory and physical qualities, with minimal impact on nutritional content. Although hydrocolloids typically have little effect on the nutritional content of gluten‐free bread, Tabrizi et al. (2023) reported that increasing HPMC levels (0.5%–1.5%) raised the ash content in gluten‐free bread formulated with quinoa flour, grass pea flour, and chestnut flour.

Recent advances in gluten‐free bread (GFB) research highlight the increasing potential of legume and pseudocereal flours as functional ingredients to improve the technological and nutritional quality of gluten‐free products. Legume flours (including white bean, soy, and cowpea) increase protein content, fiber levels, and mineral availability while also improving dough viscoelasticity and gas retention (Melini et al. 2017; Ndjang et al. 2023; Tuna et al. 2023). Similarly, pseudocereals such as amaranth, quinoa, and buckwheat improve dough rheology, loaf volume, and crumb structure, increasing consumer acceptability due to improved texture and flavor balance (Aguiar et al. 2021; Alvarez‐Jubete et al. 2010).

However, the functional performance of these flours is highly dependent on processing conditions, formulation ratios, and the presence of hydrocolloids or fermentation steps, all of which can influence starch‐protein interactions and microstructural development. Optimization studies, particularly those that incorporate rheological, physicochemical, and sensory analyses, show that combining legumes and pseudocereals can produce GFB with desirable softness, moisture retention, and sensory appeal, comparable to wheat‐based bread (Ndjang et al. 2023). Overall, a synergistic approach that combines ingredient functionality with process optimization is the most promising path forward for the next generation of nutritionally rich, technologically stable, and consumer‐acceptable gluten‐free bread.

HPMC is a non‐digestible, cellulose‐derived polymer widely used as a hydrocolloid in gluten‐free bakery formulations. Its impact on nutritional properties is primarily functional and indirect, meaning it does not contribute measurable amounts of macronutrients (protein, lipid, or carbohydrate) but rather modifies how these nutrients behave during digestion and absorption.

First, HPMC increases the apparent dietary fiber content of gluten‐free bread formulations. Although it is a synthetic derivative of cellulose and not metabolized by human enzymes, it behaves physiologically as a soluble dietary fiber. When incorporated into dough, HPMC forms a viscous matrix capable of retaining water and entrapping starch granules, thereby reducing the rate of starch gelatinization and enzymatic hydrolysis during digestion. This structural role contributes to a lower glycemic index (GI), as the slower release of glucose from starch digestion leads to moderated postprandial blood glucose responses.

Second, its strong water‐binding capacity improves the moisture retention and softness of gluten‐free bread, which can indirectly influence nutrient availability and shelf stability. By maintaining a higher water activity and softer crumb texture, HPMC can reduce starch retrogradation and protein aggregation, thus enhancing the digestibility and palatability of gluten‐free products (Sciarini et al. 2023). Finally, HPMC does not alter the quantitative composition of protein, fat, or carbohydrate in the product, as it is used in small concentrations (typically 0.5%–2% w/w of flour basis). However, it significantly affects nutrient functionality, especially starch–water and starch–protein interactions during baking and digestion, which in turn determine the product's nutritional quality and glycemic response rather than its proximate macronutrient composition.

The combination of xanthan gum (XG) and hydroxypropyl methylcellulose (HPMC) in gluten‐free bread formulations has been widely reported to produce synergistic effects on dough functionality and final product quality. XG mainly increases dough viscosity and water‐binding capacity due to its strongly anionic polysaccharide structure, which helps form a network and retain water (Lazaridou et al. 2007). In contrast, HPMC acts as a thermal gel‐forming polymer that stabilizes gas cells during baking, preventing collapse and increasing loaf volume (Rosell et al. 2001).

When used together, XG and HPMC have complementary effects: XG enhances hydration and viscosity of the dough, while HPMC improves gas‐holding capacity and crumb elasticity. These interactions lead to better dough viscoelasticity, improved gas retention during fermentation, and a superior crumb structure compared to formulations with only one hydrocolloid (Sciarini et al. 2023). Additionally, this combination helps mimic gluten's viscoelastic behavior, resulting in bread that is softer, has more uniform porosity, and stales more slowly. Therefore, using XG and HPMC together is an effective way to improve both the dough's rheology and the bread's sensory qualities.

## 4 Conclusion

The effect of hydrocolloids on gluten‐free bread depends on their concentration, the amount of water added, physicochemical properties, and interactions within the formulation. A single type of hydrocolloid may yield different results across formulations, as optimal levels are not universal. Variations in impact often arise from synergistic interactions with starch, influencing its behavior during gelatinization and retrogradation. Therefore, identifying the optimal concentration for each specific formulation is essential.

No single hydrocolloid has shown the ability to optimize all quality parameters of bread simultaneously. While the incorporation of a particular hydrocolloid may enhance certain properties, it often compromises others, thereby making the selection depend on the specific attributes being prioritized. From a consumer standpoint, desirable gluten‐free bread characteristics include increased loaf volume, a softer crumb structure, favorable flavor, appealing appearance, and prolonged shelf life. Given that the functional contribution of an individual hydrocolloid is limited, the synergistic application of multiple hydrocolloids represents a potential approach to counteract undesirable attributes in gluten‐free bread.

## Author Contributions

**Fetriyuna Fetriyuna** and **Annisa Nur Salma:** conceptualization. **Fetriyuna Fetriyuna:** methodology. **Fetriyuna Fetriyuna**, **Annisa Nur Salma**, and **Ratna Chrismiari Purwestri:** validation. **Annisa Nur Salma:** formal analysis. **Annisa Nur Salma:** investigation. **Fetriyuna Fetriyuna** and **Annisa Nur Salma:** data curation. **Fetriyuna Fetriyuna** and **Annisa Nur Salma:** writing – original draft preparation. **Fetriyuna Fetriyuna**, **Annisa Nur Salma**, and **Ratna Chrismiari Purwestri:** writing – review and editing. **Annisa Nur Salma:** visualization. **Fetriyuna Fetriyuna** and **Ratna Chrismiari Purwestri:** supervision. All authors have read and agreed to the published version of the manuscript.

## Funding

This review research was funded by *Hibah Artikel Review* Universitas Padjadjaran, Indonesia, grant number 1959/UN6.3.1/PT.00/2021.

## Ethics Statement

The authors have nothing to report.

## Conflicts of Interest

The authors declare no conflicts of interest.

## Data Availability

The data presented in this study are available on request from the corresponding author.

## References

[fsn371292-bib-0001] Abdollahzadeh, A. , M. Vazifedoost , Z. Didar , M. H. Haddadkhodaprast , and M. Armin . 2024. “Comparison of the Effect of Hydroxyl Propyl Methyl Cellulose, Pectin, and Concentrated Raisin Juice on Gluten‐Free Bread Based on Rice and Foxtail Millet Flour.” Food Science & Nutrition 12, no. 1: 439–449. 10.1002/fsn3.3741. 38268869 PMC10804086

[fsn371292-bib-0099] Achmadi, E. R. 2022. “Innovation Snack Crackers Sorghum, Challenge and Characterization Product: A review.” Journal of Food and Agricultural Product 2, no. 1: 1. 10.32585/jfap.v2i1.2148.

[fsn371292-bib-0002] Aguiar, E. V. , F. G. Santos , A. C. L. S. Centeno , and V. D. Capriles . 2021. “Influence of Pseudocereals on Gluten‐Free Bread Quality: A Study Integrating Dough Rheology, Bread Physical Properties and Acceptability.” Food Research International 150: 110762. 10.1016/j.foodres.2021.110762. 34865780

[fsn371292-bib-0003] Aguilar, N. , E. Albanell , B. Miñarro , and M. Capellas . 2015. “Chickpea and Tiger Nut Flours as Alternatives to Emulsifier and Shortening in Gluten‐Free Bread.” LWT‐Food Science and Technology 62, no. 1: 225–232. 10.1016/j.lwt.2014.12.045.

[fsn371292-bib-0004] Ahlborn, G. J. , O. A. Pike , S. B. Hendrix , W. M. Hess , and C. S. Huber . 2005. “Sensory, Mechanical, and Microscopic Evaluation of Staling in Low‐Protein and Gluten‐Free Breads.” Cereal Chemistry 82, no. 3: 328–335. 10.1094/CC-82-0328.

[fsn371292-bib-0005] Alencar, M. L. , C. L. Ortiz‐Agostinho , I. Nishitokukado , et al. 2012. “Prevalence of Celiac Disease Among Blood Donors in SÃO PAULO–The Most Populated City in Brazil.” Clinics 67, no. 9: 1013–1018. 10.6061/clinics/2012(09)05. 23018296 PMC3438239

[fsn371292-bib-0006] Alizadeh‐Bahaabadi, G. , L. Lakzadeh , H. Forootanfar , and H. R. Akhavan . 2022. “Optimization of Gluten‐Free Bread Production With Low Aflatoxin Level Based on Quinoa Flour Containing Xanthan Gum and Laccase Enzyme.” International Journal of Biological Macromolecules 200: 61–76. 10.1016/j.ijbiomac.2021.12.091. 34973985

[fsn371292-bib-0007] Alvarez‐Jubete, L. , M. Auty , E. K. Arendt , and E. Gallagher . 2010. “Baking Properties and Microstructure of Pseudocereal Flours in Gluten‐Free Bread Formulations.” European Food Research and Technology 230, no. 3: 437–445. 10.1007/s00217-009-1184-z.

[fsn371292-bib-0008] Anton, A. A. , and S. D. Artfield . 2008. “Hydrocolloids in Gluten‐Free Breads: A Review.” International Journal of Food Sciences and Nutrition 59, no. 1: 11–23. 10.1080/09637480701625630. 18097842

[fsn371292-bib-0009] Ari Akin, P. , R. Miller , T. Jaffe , K. Koppel , and L. Ehmke . 2019. “Sensory Profile and Quality of Chemically Leavened Gluten‐Free Sorghum Bread Containing Different Starches and Hydrocolloids.” Journal of the Science of Food and Agriculture 99, no. 9: 4391–4396. 10.1002/jsfa.9673. 30859568

[fsn371292-bib-0010] Baldino, N. , F. Laitano , F. R. Lupi , S. Curcio , and D. Gabriele . 2018. “Effect of HPMC and CMC on Rheological Behavior at Different Temperatures of Gluten‐Free Bread Formulations Based on Rice and Buckwheat Flours.” European Food Research and Technology 244, no. 10: 1829–1842. 10.1007/s00217-018-3096-2.

[fsn371292-bib-0012] Barcenas, M. E. , and C. M. Rosell . 2005. “Effect of HPMC Addition on the Microstructure, Quality and Aging of White Bread.” Food Hydrocolloids 19, no. 6: 1037–1043.

[fsn371292-bib-0011] Bárcenas, M. E. , and C. M. Rosell . 2006. “Different Approaches for Improving the Quality and Extending the Shelf Life of the Partially Baked Bread: Low Temperatures and Hpmc Addition.” Journal of Food Engineering 72: 92–99.

[fsn371292-bib-0013] Belorio, M. , and M. Gómez . 2020. “Effect of Hydration on Gluten‐Free Breads Made With Hydroxypropyl Methylcellulose in Comparison With Psyllium and Xanthan Gum.” Food 9, no. 11: 1548. 10.3390/foods9111548. PMC7693925 33114635

[fsn371292-bib-0014] Brouns, F. J. P. H. , V. J. Van Buul , and P. R. Shewry . 2013. “Does Wheat Make Us Fat and Sick?” Journal of Cereal Science 58, no. 2: 209–215.

[fsn371292-bib-0015] Burešová, I. , L. Masaříková , L. Hřivna , S. Kulhanová , and D. Bureš . 2016. “The Comparison of the Effect of Sodium Caseinate, Calcium Caseinate, Carboxymethyl Cellulose and Xanthan Gum on Rice‐Buckwheat Dough Rheological Characteristics and Textural and Sensory Quality of Bread.” LWT‐Food Science and Technology 68: 659–666. 10.1016/j.lwt.2016.01.010.

[fsn371292-bib-0016] Cajas Locke, J. E. , L. C. González , M. A. Loubes , and M. P. Tolaba . 2019. “Optimization of Rice Bread Formulation by Mixture Design and Relationship of Bread Quality to Flour and Dough Attributes.” LWT 113: 108299. 10.1016/j.lwt.2019.108299.

[fsn371292-bib-0017] Calle, J. , Y. Benavent‐Gil , and C. M. Rosell . 2020. “Development of Gluten Free Breads From *Colocasia esculenta* Flour Blended With Hydrocolloids and Enzymes.” Food Hydrocolloids 98: 105243. 10.1016/j.foodhyd.2019.105243.

[fsn371292-bib-0018] Chakraborty, S. K. , N. Kotwaliwale , and S. A. Navale . 2020. “Selection and Incorporation of Hydrocolloid for Gluten‐Free Leavened Millet Breads and Optimization of the Baking Process Thereof.” LWT 119: 108878. 10.1016/j.lwt.2019.108878.

[fsn371292-bib-0019] Chueamchaitrakun, P. , N. Punbusayakul , P. Wongklom , P. Chueamchaitrakun , and N. Punbusayakul . 2016. “Effect of Xanthan Gum/CMC on Bread Quality Made From Hom Nil Rice Flour.” International Food Research Journal 23, no. 5: 2300.

[fsn371292-bib-0020] Coronel, E. B. , E. N. Guiotto , M. C. Aspiroz , M. C. Tomás , S. M. Nolasco , and M. I. Capitani . 2021. “Development of Gluten‐Free Premixes With Buckwheat and Chia Flours: Application in a Bread Product.” LWT 141: 110916. 10.1016/j.lwt.2021.110916.

[fsn371292-bib-0021] Crockett, R. , P. Ie , and Y. Vodovotz . 2011. “How Do Xanthan and Hydroxypropyl Methylcellulose Individually Affect the Physicochemical Properties in a Model Gluten‐Free Dough?” Journal of Food Science 76, no. 3: E274–E282. 10.1111/j.1750-3841.2011.02088.x. 21535827

[fsn371292-bib-0022] Culetu, A. , D. E. Duta , M. Papageorgiou , and T. Varzakas . 2021. “The Role of Hydrocolloids in Gluten‐Free Bread and Pasta; Rheology, Characteristics, Staling and Glycemic Index.” Food 10, no. 12: 3121. 10.3390/foods10123121. PMC8701227 34945672

[fsn371292-bib-0023] Dapˇcevi'c‐Hadnađev, T. R. , L. P. Doki'c , M. Hadnađev , M. M. Poji'c , S. M. Rakita , and A. M. Torbica . 2013. “Changes in Quality Parameters of Bread Supplemented With OSA Starch During Storage.” Food and Feed Research 40: 101–108.

[fsn371292-bib-0024] Demirkesen, I. , S. Kelkar , O. H. Campanella , G. Sumnu , S. Sahin , and M. Okos . 2014. “Characterization of Structure of Gluten‐Free Breads by Using X‐Ray Microtomography.” Food Hydrocolloids 36: 37–44. 10.1016/j.foodhyd.2013.09.002.

[fsn371292-bib-0025] Demirkesen, I. , B. Mert , G. Sumnu , and S. Sahin . 2010. “Rheological Properties of Gluten‐Free Bread Formulations.” Journal of Food Engineering 96, no. 2: 295–303. 10.1016/j.jfoodeng.2009.08.004.

[fsn371292-bib-0026] Djordjević, M. , D. Šoronja‐Simović , I. Nikolić , M. Djordjević , Z. Šereš , and M. Milašinović‐Šeremešić . 2019. “Sugar Beet and Apple Fibres Coupled With Hydroxypropylmethylcellulose as Functional Ingredients in Gluten‐Free Formulations: Rheological, Technological and Sensory Aspects.” Food Chemistry 295: 189–197. 10.1016/j.foodchem.2019.05.066. 31174749

[fsn371292-bib-0027] Encina‐Zelada, C. R. , V. Cadavez , F. Monteiro , J. A. Teixeira , and U. Gonzales‐Barron . 2018. “Combined Effect of Xanthan Gum and Water Content on Physicochemical and Textural Properties of Gluten‐Free Batter and Bread.” Food Research International 111: 544–555. 10.1016/j.foodres.2018.05.070. 30007717

[fsn371292-bib-0028] Encina‐Zelada, C. R. , V. Cadavez , J. A. Teixeira , and U. Gonzales‐Barron . 2019. “Optimization of Quality Properties of Gluten‐Free Bread by a Mixture Design of Xanthan, Guar, and Hydroxypropyl Methyl Cellulose Gums.” Food 8, no. 5: 156. 10.3390/foods8050156. PMC6560447 31083333

[fsn371292-bib-0029] Ewerling, M. , N. C. Steinmacher , M. R. Dos Santos , et al. 2020. “Defatted Chia Flour Improves Gluten‐Free Bread Nutritional Aspects: A Model Approac.” Food Science and Technology Brazil 40: 68–75. 10.1590/fst.42118.

[fsn371292-bib-0030] Franco, V. A. , L. G. C. Garcia , and F. A. da Silva . 2020. “Addition of Hydrocolidics in Gluten‐Free Bread and Replacement of Rice Flour for Sweet Potato Flour.” Food Science and Technology Brazil 40: 88–96. 10.1590/fst.05919.

[fsn371292-bib-0031] Fujii, K. , M. Usui , A. Ohsuga , and M. Tsuji . 2023. “Effect of Thermoresponsive Xyloglucan on the Bread‐Making Properties and Preservation of Gluten‐Free Rice‐Flour Bread.” Food 12, no. 14: 2761. 10.3390/foods12142761. PMC10379171 37509853

[fsn371292-bib-0032] Gallagher, E. , T. Gormley , and E. Arendt . 2003. “Crust and Crumb Characteristics of Gluten Free Breads.” Journal of Food Engineering 56, no. 2–3: 153–161. 10.1016/S0260-8774(02)00244-3.

[fsn371292-bib-0033] Gallagher, E. , T. Gormley , and E. Arendt . 2004. “Recent Advances in the Formulation of Gluten‐Free Cereal‐Based Products.” Trends in Food Science & Technology 15, no. 3–4: 143–152. 10.1016/j.tifs.2003.09.012.

[fsn371292-bib-0034] Golley, S. , N. Corsini , D. Topping , M. Morell , and P. Mohr . 2014. “Motivations for Avoiding Wheat Consumption in Australia: Results From a Population Survey.” Public Health Nutrition 18, no. 3: 490–499. 24739252 10.1017/S1368980014000652 PMC10271735

[fsn371292-bib-0035] Green, P. H. R. , and C. Cellier . 2007. Celiac Disease. Columbia University College of Physicians and Surgeons.

[fsn371292-bib-0036] Guarda, A. , C. M. Rosell , C. Benedito , and M. J. Galotto . 2004. “Different Hydrocolloids as Bread Improvers and Antistaling Agents.” Food Hydrocolloids 18: 241–247.

[fsn371292-bib-0037] Hager, A.‐S. , and E. K. Arendt . 2013. “Influence of Hydroxypropylmethylcellulose (Hpmc), Xanthan Gum and Their Combination on Loaf Specific Volume, Crumb Hardness and Crumb Grain Characteristics of Gluten‐Free Breads Based on Rice, Maize, Teff and Buckwheat.” Food Hydrocolloids 32: 195–203.

[fsn371292-bib-0038] Harris, C. 2011. “Two of A Kind: Celiac Disease and Thyroid Disease. Today's Dietitian.”

[fsn371292-bib-0039] Hernández‐Aguirre, M. A. , J. J. Islas‐Hernández , M. E. Sánchez‐Pardo , S. L. Rodríguez‐Ambriz , and P. Osorio‐Díaz . 2019. “Response Surface Methodology for Optimization of Gluten‐Free Bread Made With Unripe Banana Flour.” Journal of Food Measurement and Characterization 13, no. 3: 1652–1660. 10.1007/s11694-019-00082-y.

[fsn371292-bib-0041] Horstmann, S. W. , C. Axel , and E. K. Arendt . 2018. “Water Absorption as a Prediction Tool for the Application of Hydrocolloids in Potato Starch‐Based Bread.” Food Hydrocolloids 81: 129–138. 10.1016/j.foodhyd.2018.02.045.

[fsn371292-bib-0040] Horstmann, S. , M. Belz , M. Heitmann , E. Zannini , and E. Arendt . 2016. “Fundamental Study on the Impact of Gluten‐Free Starches on the Quality of Gluten‐Free Model Breads.” Food 5, no. 2: 30. 10.3390/foods5020030. PMC5302342 28231125

[fsn371292-bib-0042] Huerta, K. D. A. M. , J. Alves , S. Dos , A. F. C. D. A. Silva , E. H. Kubota , and C. S. D. A. Rosa . 2016. “Sensory Response and Physical Characteristics of Gluten‐Free and Gum‐Free Bread With Chia Flour.” Food Science and Technology 36, no. suppl 1: 15–18. 10.1590/1678-457x.0032.

[fsn371292-bib-0043] Imamura, M. , S. Ito , and E. Arai . 2021. “Effects of Different Types of Hydrocolloids on the Quality Improvement of Gluten‐Free Rice Flour Bread Made With Soymilk.” Food Science and Technology Research 27, no. 3: 389–395. 10.3136/fstr.27.389.

[fsn371292-bib-0044] Jang, K. , Y. E. Jin Hong , Y. Moon , S. Jeon , S. Angalet , and M. Kweon . 2018. “Exploring the Applicability of Tamarind Gum for Making Gluten‐Free Rice Bread.” Food Science and Biotechnology 27, no. 6: 1639–1648. 10.1007/s10068-018-0416-z. 30483427 PMC6233388

[fsn371292-bib-0045] Kang, M. Y. , Y. H. Choi , and H. C. Choi . 1997. “Effects of Gums, Fats and Glutens Adding on Processing and Quality of Milled Rice Bread.” Korean Journal of Food Science and Technology 29: 700–704.

[fsn371292-bib-0046] Katina, K. , M. Salmenkallio‐Marttila , R. Partanen , P. Forssell , and K. Autio . 2006. “Effects of Sourdough and Enzymes on Staling of High‐Fibre Wheat Bread.” LWT ‐ Food Science and Technology 39, no. 5: 479–491. 10.1016/j.lwt.2005.03.013.

[fsn371292-bib-0047] Kittisuban, P. , P. Ritthiruangdej , and M. Suphantharika . 2014. “Optimization of Hydroxypropylmethylcellulose, Yeast β‐Glucan, and Whey Protein Levels Based on Physical Properties of Gluten‐Free Rice Bread Using Response Surface Methodology.” LWT‐Food Science and Technology 57, no. 2: 738–748. 10.1016/j.lwt.2014.02.045.

[fsn371292-bib-0048] Krupa‐Kozak, U. , N. Baczek , and C. M. Rosell . 2013. “Application of Dairy Proteins as Technological and Nutritional Improvers of Calcium‐Supplemented Gluten‐Free Bread.” Nutrients 5, no. 11: 4503–4520. 10.3390/nu5114503. 24241213 PMC3847745

[fsn371292-bib-0049] Lazaridou, A. , D. Duta , M. Papageorgiou , N. Belc , and C. G. Biliaderis . 2007. “Effects of Hydrocolloids on Dough Rheology and Bread Quality Parameters in Gluten‐Free Formulations.” Journal of Food Engineering 79, no. 3: 1033–1047. 10.1016/j.jfoodeng.2006.03.032.

[fsn371292-bib-0100] Li, X. , S. Al‐Assaf , Y. Fang , and G. O. Phillips . 2013. “Competitive Adsorption between Sugar Beet Pectin (Sbp) and Hydroxypropyl Methylcellulose (HPMC) at the Oil/Water Interface.” Carbohydrate Polymers 91: 573–580. 23121947 10.1016/j.carbpol.2012.08.075

[fsn371292-bib-0050] Linlaud, N. , E. Ferrer , M. C. Puppo , and C. Ferrero . 2011. “Hydrocolloid Interaction With Water, Protein, and Starch in Wheat Dough.” Journal of Agricultural and Food Chemistry 59, no. 2: 713–719. 10.1021/jf1026197. 21175189

[fsn371292-bib-0051] Liu, X. , T. Mu , H. Sun , M. Zhang , J. Chen , and M. L. Fauconnier . 2018. “Influence of Different Hydrocolloids on Dough Thermo‐Mechanical Properties and In Vitro Starch Digestibility of Gluten‐Free Steamed Bread Based on Potato Flour.” Food Chemistry 239: 1064–1074. 10.1016/j.foodchem.2017.07.047. 28873523

[fsn371292-bib-0052] Mancebo, C. M. , M. M. Martínez , C. Merino , E. de la Hera , and M. Gómez . 2017. “Effect of Oil and Shortening in Rice Bread Quality: Relationship Between Dough Rheology and Quality Characteristics.” Journal of Texture Studies 48, no. 6: 597–606. 10.1111/jtxs.12270. 28449185

[fsn371292-bib-0053] Mariani, P. , M. G. Viti , M. Montouri , et al. 1998. “The Gluten‐Free Diet: A Nutritional Risk Factor for Adolescents With Celiac Disease?” Journal of Pediatric Gastroenterology and Nutrition 27, no. 5: 519–523. 10.1097/00005176-199811000-00004. 9822315

[fsn371292-bib-0054] Martínez, M. M. , and M. Gómez . 2017. “Rheological and Microstructural Evolution of the Most Common Gluten‐Free Flours and Starches During Bread Fermentation and Baking.” Journal of Food Engineering 197: 78–86. 10.1016/j.jfoodeng.2016.11.008.

[fsn371292-bib-0055] Masih, J. , and A. Sharma . 2016. “Study on Consumer Behaviour and Economic Advancements of Gluten‐Free Products.” American Journal of Experimental Agriculture 12, no. 1: 1–10. 10.9734/ajea/2016/24737.

[fsn371292-bib-0056] Matia‐Merino, L. , M. Prieto , L. Roman , and M. Gómez . 2019. “The Impact of Basil Seed Gum on Native and Pregelatinized Corn Flour and Starch Gel Properties.” Food Hydrocolloids 89: 122–130. 10.1016/j.foodhyd.2018.10.005.

[fsn371292-bib-0057] McCarroll, L. , and S. de Kock . 2017. “Evaluation of Mageu‐Based Gluten‐Free Bread Loaf Characteristics.” African Journal of Science, Technology, Innovation and Development 9, no. 2: 147–156. 10.1080/20421338.2017.1296076.

[fsn371292-bib-0058] Melini, F. , V. Melini , F. Luziatelli , and M. Ruzzi . 2017. “Current and Forward‐Looking Approaches to Technological and Nutritional Improvements of Gluten‐Free Bread With Legume Flours: A Critical Review.” Comprehensive Reviews in Food Science and Food Safety 16, no. 5: 1101–1122. 10.1111/1541-4337.12279. 33371611

[fsn371292-bib-0059] Mir, S. A. , M. A. Shah , H. R. Naik , and I. A. Zargar . 2016. “Influence of Hydrocolloids on Dough Handling and Technological Properties of Gluten‐Free Breads.” Trends in Food Science & Technology 51: 49–57. 10.1016/j.tifs.2016.03.005.

[fsn371292-bib-0060] Mohammadi, M. , N. Sadeghnia , M.‐H. Azizi , T.‐R. Neyestani , and A. M. Mortazavian . 2014. “Development of Gluten‐Free Flat Bread Using Hydrocolloids: Xanthan and CMC.” Journal of Industrial and Engineering Chemistry 20, no. 4: 1812–1818. 10.1016/j.jiec.2013.08.035.

[fsn371292-bib-0061] Muna, S. N. , S. Noviasari , and M. Muzaifa . 2023. “Pangan Lokal Sebagai Bahan Baku Produk Bakeri Non‐Gluten Ulasan Jenis Dan Karakteristik Produk Yang Dihasilkan.” Jurnal Ilmiah Mahasiswa Pertanian 8, no. 3: 345–351.

[fsn371292-bib-0062] Naji‐Tabasi, S. , and M. Mohebbi . 2015. “Evaluation of Cress Seed Gum and Xanthan Gum Effect on Macrostructure Properties of Gluten‐Free Bread by Image Processing.” Journal of Food Measurement and Characterization 9, no. 1: 110–119. 10.1007/s11694-014-9216-1.

[fsn371292-bib-0063] Ndjang, M. M. N. , J. M. Klang , B. Njapndounke , et al. 2023. “Optimization of the Processing Conditions for the Production of a Gluten‐Free Bread From Sour Cassava Starch ( *Manihot esculenta* ) and Some Legumes ( *Arachis hypogaea* , Vigna Unguiculata, and *Glycine max* ).” Food 12, no. 17: 3180. 10.3390/foods12173180. PMC10486541 37685113

[fsn371292-bib-0064] Nishita, K. D. , R. L. Roberts , M. M. Bean , and B. M. Kennedy . 1976. “Development of a Yeast‐Leavened Rice‐Bread Formula [Hydroxypropyl‐Methylcelluloses].” Cereal Chemistry 53: 626–635.

[fsn371292-bib-0065] Numfon, R. 2017. “Effects of Different Hydrocolloids on Properties of Gluten‐Free Bread Based on Small Broken Rice Berry Flour.” Food Science and Technology International 23, no. 4: 310–317. 10.1177/1082013217690064. 28118742

[fsn371292-bib-0066] Nunes, M. H. B. , L. A. M. Ryan , and E. K. Arendt . 2009. “Effect of Low Lactose Dairy Powder Addition on the Properties of Gluten‐Free Batters and Bread Quality.” European Food Research and Technology 229, no. 1: 31–41. 10.1007/s00217-009-1023-2.

[fsn371292-bib-0067] Okoli, C. , and K. Schabram . 2011. “A Guide to Conducting Literature Review of Information System Research.” Communications of the Association for Information Systems 37, no. 43: 879–910.

[fsn371292-bib-0068] Ozturk, O. K. , and B. Mert . 2018. “The Use of Microfluidization for the Production of Xanthan and Citrus Fiber‐Based Gluten‐Free Corn Breads.” LWT 96: 34–41. 10.1016/j.lwt.2018.05.025.

[fsn371292-bib-0069] Pereyra Gonzales, A. S. , G. B. Naranjo , G. E. Leiva , and L. S. Malec . 2010. “Maillard Reaction Kinetics in Milk Powder: Effect of Water Activity at Mild Temperatures.” International Dairy Journal 20, no. 1: 40–45. 10.1016/j.idairyj.2009.07.007.

[fsn371292-bib-0070] Pérez‐Quirce, S. , C. Collar , and F. Ronda . 2014. “Significance of Healthy Viscous Dietary Fibres on the Performance of Gluten‐Free Rice‐Based Formulated Breads.” International Journal of Food Science and Technology 49, no. 5: 1375–1382. 10.1111/ijfs.12439.

[fsn371292-bib-0071] Pico, J. , M. P. Reguilón , J. Bernal , and M. Gómez . 2019. “Effect of Rice, Pea, Egg White and Whey Proteins on Crust Quality of Rice Flour‐Corn Starch Based Gluten‐Free Breads.” Journal of Cereal Science 86: 92–101. 10.1016/j.jcs.2019.01.014.

[fsn371292-bib-0072] Pomeranz, Y. 1960. “Determination of Bread Crumb Color as Related to the Color of Flour Used to Bake the Bread.” Cereal Chemistry 37: 765–772.

[fsn371292-bib-0073] Rakkhumkaew, N. , Y. Boonsri , and A. Sukchum . 2019. “Utilization of Small Broken Riceberry Flour in Gluten‐Free Bread.” Food Science and Technology International 25, no. 6: 515–522. 10.1177/1082013219842463. 30971119

[fsn371292-bib-0074] Reilly, N. R. 2016. “The Gluten‐Free Diet: Recognising Fact, Fiction, and Fad.” Journal of Pediatrics 175: 206–210. 27185419 10.1016/j.jpeds.2016.04.014

[fsn371292-bib-0075] Rosell, C. M. , J. A. Rojas , and C. de Benedito Barber . 2001. “Influence of Hydrocolloids on Dough Rheology and Bread Quality.” Food Hydrocolloids 21: 452–462.

[fsn371292-bib-0076] Rustagi, S. , S. Khan , S. Choudhary , et al. 2018. “Hydroxypropyl Methylcellulose and Whey Protein Concentrate as Technological Improver in Formulation of Gluten‐Free Protein Rich Bread.” Current Research in Nutrition and Food Science Journal 6, no. 1: 211–221. 10.12944/CRNFSJ.6.1.24.

[fsn371292-bib-0077] Sabanis, D. , D. Lebesi , and C. Tzia . 2009a. “Development of Fibre‐Enriched Gluten‐Free Bread: A Response Surface Methodology Study.” International Journal of Food Sciences and Nutrition 60, no. sup4: 174–190. 10.1080/09637480902721196. 19330631

[fsn371292-bib-0078] Sabanis, D. , D. Lebesi , and C. Tzia . 2009b. “Effect of Dietary Fibre Enrichment on Selected Properties of Gluten‐Free Bread.” LWT‐Food Science and Technology 42, no. 8: 1380–1389. 10.1016/j.lwt.2009.03.010.

[fsn371292-bib-0079] Sabanis, D. , and C. Tzia . 2011. “Effect of Hydrocolloids on Selected Properties of Gluten‐Free Dough and Bread.” Food Science and Technology International 17, no. 4: 279–291. 10.1177/1082013210382350. 21917639

[fsn371292-bib-0080] Sabanis, D. , C. Tzia , and S. Papadakis . 2008. “Effect of Different Raisin Juice Preparations on Selected Properties of Gluten‐Free Bread.” Food and Bioprocess Technology 1, no. 4: 374–383. 10.1007/s11947-007-0027-9.

[fsn371292-bib-0081] Sadat, A. , W. Cao , M. Sharma , L. Duizer , and I. J. Joye . 2023. “Enhancing Zein‐Starch Dough and Bread Properties by Addition of Hydrocolloids.” Food Hydrocolloids 143: 108860. 10.1016/j.foodhyd.2023.108860.

[fsn371292-bib-0101] Sarkar, N. , and L. C. Walker . 1995. “Hydration—Dehydration Properties of Methylcellulose Andhydroxypropylmethylcellulose.” Carbohydrate Polymers 27, no. 3: 177–185. 10.1016/0144-8617(95)00061-B.

[fsn371292-bib-0082] Sasaki, T. 2018. “Effects of Xanthan and Guar Gums on Starch Digestibility and Texture of Rice Flour Blend Bread.” Cereal Chemistry 95, no. 1: 177–184. 10.1002/cche.10024.

[fsn371292-bib-0083] Sciarini, L. S. , P. M. Palavecino , P. D. Ribotta , and G. N. Barrera . 2023. “ *Gleditsia triacanthos* Galactomannans in Gluten‐Free Formulation: Batter Rheology and Bread Quality.” Food 12, no. 4: 756. 10.3390/foods12040756. PMC9956313 36832831

[fsn371292-bib-0084] Srikanlaya, C. , N. Therdthai , P. Ritthiruangdej , and W. Zhou . 2018. “Effect of Hydroxypropyl Methylcellulose, Whey Protein Concentrate and Soy Protein Isolate Enrichment on Characteristics of Gluten‐Free Rice Dough and Bread.” International Journal of Food Science and Technology 53, no. 7: 1760–1770. 10.1111/ijfs.13761.

[fsn371292-bib-0085] Stea, S. , and G. J. Pickering . 2019. “Optimizing Messaging to Reduce Red Meat Consumption.” Environmental Communication 13, no. 5: 633–648. 10.1080/17524032.2017.1412994.

[fsn371292-bib-0086] Surono, D. I. , I. E. J. Nurali , and I. J. S. Moningka . 2017. “Kualitas Fisik Dan Sensoris Roti Tawar Bebas Gluten Bebas Kasein Berbahan Dasar Tepung Komposit Pisang Goroho (*Musa acuminate* L).” Cocos 8, no. 2: 1–12. 10.35791/cocos.v1i1.14852.

[fsn371292-bib-0087] Sutrisno, A. , S. S. Yuwono , and I. Ikarini . 2021. “Effect of Glucomannan and Xanthan Gum Proportion on the Physical and Sensory Characteristic of Gluten‐Free Bread.” IOP Conference Series: Earth and Environmental Science 924, no. 1: 012028. 10.1088/1755-1315/924/1/012028.

[fsn371292-bib-0088] Sworn, G. 2009. “Xanthan Gum.” In Handbook of Hydrocolloids, 186–203. Elsevier. 10.1533/9781845695873.186.

[fsn371292-bib-0089] Tabrizi, A. S. , E. Ataye Salehi , A. Arianfar , and Z. Sheikholesalami . 2023. “Impact of Cress Seed and Basil Gum and HPMC on Physicochemical and Textural Properties of Gluten‐Free Bread.” Journal of Agricultural Science and Technology 25, no. 1: 75–86. 10.52547/jast.25.1.75.

[fsn371292-bib-0090] Tamilselvan, T. , S. Sharma , P. E. Thomas , K. Goyal , and P. Prabhasankar . 2022. “Role of Hydrocolloids in Improving the Rheology, Quality Characteristics, and Microstructure of Gluten‐Free Proso Millet Bread.” International Journal of Food Science and Technology 57, no. 11: 7156–7166. 10.1111/ijfs.16058.

[fsn371292-bib-0091] Tufaro, D. , A. Bassoli , and C. Cappa . 2022. “Okra ( *Abelmoschus esculentus* ) Powder Production and Application in Gluten‐Free Bread: Effect of Particle Size.” Food and Bioprocess Technology 15, no. 4: 904–914. 10.1007/s11947-022-02784-6.

[fsn371292-bib-0092] Tuna, A. , C. Cappa , F. Tokatli , and C. Alamprese . 2023. “White Bean and Hazelnuts Flours: Application in Gluten‐Free Bread.” LWT 184: 114995. 10.1016/j.lwt.2023.114995.

[fsn371292-bib-0093] Tunç, M. T. , and T. Kahyaoglu . 2016. “Improving Rheological and Baking Properties of Gluten‐Free Breads Using Defatted Hazelnut Flour With Various Gums.” International Journal of Food Engineering 12, no. 4: 343–353. 10.1515/ijfe-2015-0207.

[fsn371292-bib-0094] Vidaurre‐Ruiz, J. , S. Matheus‐Diaz , F. Salas‐Valerio , G. Barraza‐Jauregui , R. Schoenlechner , and R. Repo‐Carrasco‐Valencia . 2019. “Influence of Tara Gum and Xanthan Gum on Rheological and Textural Properties of Starch‐Based Gluten‐Free Dough and Bread.” European Food Research and Technology 245, no. 7: 1347–1355. 10.1007/s00217-019-03253-9.

[fsn371292-bib-0095] Xiao, Y. , and M. Watson . 2019. “Guidance on Conducting a Systematic Literature Review.” Journal of Planning Education and Research 39, no. 1: 93–112. 10.1177/0739456X17723971.

[fsn371292-bib-0096] Zarringhalami, S. , A. Ganjloo , and Z. Mokhtari Nasrabadi . 2021. “Optimization Xanthan Gum, Roselle Seed and Egg White Powders Levels Based on Textural and Sensory Properties of Gluten‐Free Rice Bread.” Journal of Food Science and Technology 58, no. 3: 1124–1131. 10.1007/s13197-020-04626-9. 33678894 PMC7884577

[fsn371292-bib-0097] Zhao, F. , Y. Li , C. Li , et al. 2021. “Co‐Supported Hydrocolloids Improve the Structure and Texture Quality of Gluten‐Free Bread.” LWT 152: 112248. 10.1016/j.lwt.2021.112248.

[fsn371292-bib-0098] Ziobro, R. , T. Witczak , L. Juszczak , and J. Korus . 2013. “Supplementation of Gluten‐Free Bread With Non‐Gluten Proteins. Effect on Dough Rheological Properties and Bread Characteristic.” Food Hydrocolloids 32, no. 2: 213–220. 10.1016/j.foodhyd.2013.01.006.

